# Health experiences and outcomes of autistic and non-autistic adults with hypermobile Ehlers-Danlos syndrome and hypermobility spectrum disorder

**DOI:** 10.1186/s12916-026-04713-2

**Published:** 2026-02-25

**Authors:** Catherine J. Crompton, Themis N. Efthimiou, Dervil M. Dockrell, Kathryn M. Berg

**Affiliations:** 1 Usher Institute, University of Edinburgh, Edinburgh, UK; 2 Centre for Clinical Brain Sciences, University of Edinburgh, Edinburgh, UK; 3 Institute of Genetics and Cancer, University of Edinburgh, Edinburgh, UK

**Keywords:** Autism, Hypermobility, Hypermobility spectrum disorder, Hypermobile Ehlers-Danlos syndrome

## Abstract

**Background:**

Previous research has indicated an association between hypermobility and autism. This study examined whether being autistic affects diagnosis, symptoms, and health experiences of people with hypermobility, including hypermobile Ehlers-Danlos syndrome (hEDS) and hypermobility spectrum disorders (HSD). We compare three groups: autistic adults, non-autistic adults, and adults who are not autistic but have a high level of autistic traits. Additionally, we examined which health and social factors predicted self-reported physical and mental health outcomes for autistic and non-autistic people with hEDS/HSD.

**Method:**

A total of 1754 participants completed an online questionnaire about their diagnostic experience, hypermobile symptoms, co-occurring health conditions, self-rated physical and mental health, engagement with health services, and employment and benefits status. Around 25% of respondents were autistic; a further 25% had high levels of autistic traits.

**Results:**

Autistic participants had more symptoms of hEDS/HSD and were more likely to have co-occurring physical and mental health conditions, compared with non-autistic and high autistic trait respondents. Autistic and high autistic trait participants self-reported poorer physical and mental health compared with non-autistic participants. Autistic participants’ mental health was impacted by limitations to everyday activities, whereas non-autistic participants were more impacted by difficulties with self-care.

**Conclusions:**

Autistic and non-autistic people may experience hEDS/HSD differently, which may impact the type of supports that are most beneficial to them.

**Supplementary Information:**

The online version contains supplementary material available at 10.1186/s12916-026-04713-2.

## Background

Hypermobility spectrum disorders (HSD) and hypermobile Ehlers-Danlos syndrome (hEDS) are hereditary, heterogenous connective tissue disorders most commonly characterised by joint hypermobility and tissue fragility [10, 65]. At present, there is no genetic test for HSD or hEDS. A diagnosis of hEDS is based on clinical assessment examining: (1) the presence of joint hypermobility; (2) the presence of other systemic physical symptoms and musculoskeletal complications, (3) family history; and (4) the exclusion of other acquired or heritable connective tissue disorders [74]. Common symptoms of joint hypermobility include stiffness, clicking joints, fatigue, and recurrent injuries [64]. Though not included in the 2017 diagnostic criteria, symptoms associated with other systemic issues can be wide ranging, including extra-articular symptoms such chronic pain, fatigue, orthostatic intolerance, gastrointestinal disorders, and pelvic and bladder dysfunction. People who do not meet the specific diagnostic criteria for hEDS but experience joint hypermobility and systemic symptoms may be given a diagnosis of HSD [2].

The two conditions are often researched jointly given the overlap in diagnostic pathways and management [3, 37]. The prevalence of hEDS and HSD is estimated between 1.2 in 1000 [14] and 4.4 in 1000 [58]; however, these figures may be underestimated as many people with hEDS and HSD face significant delays to diagnosis or are misdiagnosed with another condition [8]. When assessing patients for hypermobility, clinicians may use the Beighton score, a nine-point scale to assess joint flexibility; however, the tools’ sensitivity to accurately detect joint hypermobility remains controversial as its assessment focuses predominantly on the upper limbs and does not consider age- and sex-related differences in tissue laxity [43]. The lack of genetic tests for hEDS/HSD, the wide-ranging symptoms and co-occurring conditions experienced, and a wide spectrum of severity can result in a lengthy diagnostic trajectory [3, 61]. There are no specific treatments for hEDS/HSD, though patients can be supported to manage their symptoms through physiotherapy, pain management, medication for co-occurring conditions, mobility aids, and occupational therapy [51].

People with hEDS/HSD report poorer health-related quality of life and lower satisfaction with healthcare than those without symptomatic hypermobility [23]. A large mixed methods study found that the most common desired change to improve healthcare was improved knowledge of hEDS/HSD among healthcare professionals [23], which is currently low: a study of occupational therapists and physiotherapists in Scotland found that less than one third had received external hypermobility-specific training [20].

Autism is a neurodevelopmental condition with diagnostic criteria including differences in social communication, interaction, and restricted and repetitive behaviours [1]. Autistic people present with significantly elevated rates of a myriad of physical health conditions including auto-immune conditions, epilepsy, and atopy [34, 50, 56, 68],mental health conditions including anxiety and depression [33]; and elevated levels of pain [73]. Recently, there has been a focus on the co-occurrence of autism and hypermobility [17]. A systematic review indicated that there are increased rates of hypermobility in autistic people compared with non-autistic people—estimated between 28 and 39% [3]. Additionally, there are increased incidence of autism in symptomatic hypermobile patients and higher scores on autistic traits in symptomatic hypermobile people [3].

Both autism and hEDS/HSD are complex polygenic conditions, clinically heterogeneous, and highly hereditary [3, 13, 63]. Population-level research that suggests a link between the two exists [14], and a potential genetic link is indicated by a study which found that more than 20% of mothers with hEDS/HSD went on to have an autistic child, which is a similar rate to that of autistic mothers [11, 12]. There is also significant overlap in the experiences of autistic people and people with hEDS/HSD, including differences in sensory processing, differences in movement and co-ordination, autonomic and immune dysregulation, and increased experience of poor mental health [11, 12]. Recent work has posited links between hypermobility, proprioceptive differences, and emotional dysregulation in neurodivergent people [22]. Understanding the experiences of these groups and the barriers they face to health and social care provision is crucial to minimise negative health and wellbeing outcomes.

Autistic people have poorer health experiences and health outcomes than non-autistic people [21, 72] and face significant barriers in accessing clinical services. These include patient-level, autism-related factors such as verbal communication style, differences in non-verbal communication, sensory sensitivities, differences in bodily awareness and processing of proprioceptive input, and slower processing speeds [48]. Additionally, clinical and system-level factors can negatively affect autistic peoples’ access to care, including the sensory environment, wait time, and lack of staff knowledge and understanding of autism [45]. Autistic people self-report lower healthcare self-efficacy and lower chronic condition management self-efficacy than non-autistic people [49].

Additionally, people who have a high level of autistic traits but who are not diagnosed or self-identified as autistic may have different profiles of cognitive processing, behaviour, and language to those with non-autistic people with a low level of autistic traits [42, 59]. Hypermobile people may have higher levels of autistic traits [3]. Therefore, it is important to understand more about how those with a high level of autistic traits without a diagnosis of autism experience hEDS/HSD, care and support, and outcomes.

The link between autism, autistic traits, and hypermobility is well established, but research has not yet explored whether autistic people experience hypermobility in a similar way to non-autistic people. Additionally, we do not know if the same factors influence self-reported health and mental health in people with hEDS/HSD depending on whether they are autistic or not. In this research, we examine (1) whether autistic people, people with high levels of autistic traits, and people with low levels of autistic traits are impacted by hEDS/HSD in similar or different ways; and (2) which factors predict self-reported physical and mental health in people with hEDS/HSD who are autistic, who have high levels of autistic traits, and those who have low levels of autistic traits.

## Method

This study used a quantitative cross-sectional survey to investigate the experiences of adults with hEDS/HSD in the UK. Ethical approval was granted by the University of Edinburgh Medical Research Ethics Committee (Ref 23 – EMREC – 015).

### Patient and public involvement and engagement

Before launching the study, the questionnaire was presented to a patient steering group of representatives from Ehlers-Danlos Support UK (EDS UK), who provided feedback on the design and questions included. Before consulting with them, the intention was to only recruit participants with hEDS; however, based on their feedback, this was extended to also include participants with HSD due to the significant overlap in diagnostic pathways and condition management.

### Materials and procedure

Participants provided informed consent, before confirming they had a diagnosis of hEDS/HSD, were aged over 16, and currently lived in the UK. Participants were then asked to provide demographic information (e.g. age bracket, gender, ethnicity) and information about their diagnosis of hEDS/HSD (e.g. when diagnosis was received and from what clinical service, length of time between symptom onset and diagnosis, and their Beighton score). They were asked about their management of symptoms of hEDS/HSD (e.g. support from medical teams, pain management, and use of equipment); the psychosocial impact of hEDS/HSD; and information about other health conditions, including experiencing a range of hEDS/HSD symptoms and common co-occurring conditions (as defined in [51]). Participants self-reported if they had received a diagnosis of autism from a clinician. The full questionnaire is available in Additional file 1. It took around 20 min to complete, and participants were not reimbursed for their time.

Participants also completed three standardised measures. The first was the Autistic Spectrum Quotient (AQ), a self-reported questionnaire to measure autistic traits relating to social skills, attention switching, attention to detail, communication, and imagination [5]. The AQ is a screening tool rather than a diagnostic tool. Participants respond to 50 items using a 4-item Likert scale. The maximum score is 50, with higher scores indicating a higher level of autistic traits,a score of 32 or more typically indicates a high level of autistic traits [5].

Participants then completed the Patient Health Questionnaire-4 (PHQ-4), a self-reported measure of anxiety and depression. Respondents rate each of the items on a 4-point scale indicating how much a symptom has bothered them over the past fortnight. The maximum score is 12, with higher scores indicating a higher frequency of symptoms associated with depression and anxiety. Finally, participants completed the EuroQol (EQ-5D-5L) measure [19], at the end of which participants were asked to ‘*rate your health score today, with 0 representing the worst health you can imagine and 100 representing the best health you can imagine*’. Responses were given on a 100-point visual analogue scale (converted to a 0–1 for analysis), which included three anchor points: 0, 50, and 100.

### Participants

Participants were recruited via advertisements on the Ehlers-Danlos Support UK (EDS UK) website and via EDS UK social media. Data were collected between September 2023 and January 2024.

In total, 2771 participants took part. After removing duplicate entries (*n* = 127), participants who were ineligible due to either being under 16 years of age, or not having a clinical diagnosis of either hEDS or HSD (*n* = 440), participants who completed < 90% of the survey (*n* = 125), and participants who did not complete the AQ (*n* = 345), the final sample comprised 1754 participants. Participants were grouped based on (a) whether they had a clinical diagnosis of autism and (b) their score on the AQ. Participants in the ‘autistic group’ reported having received a clinical diagnosis of autism (*n* = 436); participants in the ‘high AQ’ group did not report a clinical diagnosis of autism though scored 32 or more on the AQ (*n* = 431); and the ‘non-autistic group’ did not have a clinical diagnosis of autism and scored below 32 on the AQ (*n* = 887).

### Statistical analyses

All analyses were conducted using R (version 4.4.2; [52]). Group comparisons for categorical variables were performed using the chi-squared test of independence. Effect sizes were calculated as Cramér’s *V* using the *vcd* package [47]. In instances where the assumptions of the chi-squared test were not met (i.e. where any cell had an expected count below 5), Fisher’s exact test was used instead. If the omnibus test indicated significant differences (*a* = 0.05), then post hoc pairwise comparisons were conducted to identify which groups differed. We used the *rcompanion* package [44] to perform pairwise chi-squared tests, with *p* values adjusted for multiple comparisons using the Bonferroni method.

For statistical modelling, the choice of model depended on the outcome variable. For the self-reported mental health (PHQ), a standard linear regression was employed. For the self-reported physical health (EuroQol), we compared several models to identify the best fit: a standard linear regression, a beta regression implemented with the *betareg* package [16], and a zero–one inflated beta model using the flexible families available in the *glmmTMB* package [9, 46]. As a sensitivity analysis, a robust linear regression from the *MASS* package [67] was also fitted to check for the influence of outliers; as this model did not improve fit and yielded similar estimates to the standard linear model, it was not used for final inference. The Akaike Information Criterion (AIC) was used to compare and select the best-fitting model from these candidates. For significant predictors in the final selected models, post hoc comparisons were performed by calculating estimated marginal means using the *emmeans* package [41], with *p* values adjusted for multiple comparisons via the Bonferroni correction method. To perform variable selection, lasso regression was conducted using the *glmnet* package [26], and *p* values for the selected predictors were adjusted to account for the selection process using the *selectiveInference* package [62].

## Results

### Participant characterisation

Participant demographics, including age, gender, and ethnicity, are presented in Table 1. Though all three groups spanned a wide age range, there was a significant difference between the three (*χ*^2^(14, *N* = 1754) = 118.00, *p* < 0.001), though with a moderate effect size (Cramér’s *V* = 0.183), indicating that while age distributions differ, those differences are not overly large. In the autistic group, more participants were in the 16–24 bracket (32.1%) compared with the non-autistic group (11.5%). The high AQ group had the highest proportion in the 25–34 range (33.9%), while the non-autistic group skewed older, with nearly 19% in the 45–54 bracket.

**Table 1:** Participant demographics by group, presented with % (*n*)

Characteristic	Overall *N* = 1754	Non-autistic *N* = 887	High AQ *N* = 431	Autistic *N* = 436
Age group				
16–24	18% (319)	11% (102)	18% (77)	32% (140)
25–34	31% (549)	30% (266)	34% (146)	31% (137)
35–44	25% (446)	28% (251)	26% (112)	19% (83)
45–54	17% (299)	19% (165)	18% (76)	13% (58)
55–64	6% (111)	9% (79)	4% (18)	3% (14)
65–74	1% (24)	2% (20)	0% (2)	0% (2)
75 +	0% (2)	0% (2)	0% (0)	0% (0)
No answer	0% (4)	0% (2)	0% (0)	0% (2)
Gender				
Female	90% (1574)	97% (861)	92% (395)	73% (318)
Male	3% (53)	2% (15)	2% (10)	6% (28)
Non-binary	7% (122)	1% (9)	6% (26)	20% (87)
No answer	0% (5)	0% (2)	0% (0)	1% (3)
Ethnicity				
Asian/Asian British/Asian Welsh	1% (11)	1% (9)	0% (1)	0% (1)
Black/Black British/Black Welsh/Caribbean/African	0% (5)	0% (3)	0% (1)	0% (1)
Mixed/multiple	3% (45)	3% (27)	2% (9)	2% (9)
No answer	5% (94)	6% (53)	5% (22)	4% (19)
Other ethnic group	1% (18)	1% (8)	1% (3)	2% (7)
White	90% (1581)	89% (787)	92% (395)	92% (399)

The three groups also differed on gender (*χ*^2^(4, *N* = 1749) = 193.74, *p* < 0.001), though again the effect size was moderate (Cramér’s *V* = 0.235). The autistic group had proportionally more non-binary (20.1%) and male (6.5%) participants than the other groups, which were predominantly female.

Participants in the autistic group had a mean AQ score of 34.71 (SD = 5.86), in the high AQ group had a mean AQ score of 37.20 (SD = 3.89), and in the non-autistic group had a mean AQ score of 20.58 (SD = 7.07).

### Diagnosis of hEDS/HSD

Of the 1754 participants in the sample, 1530 (87.23%) reported a confirmed diagnosis of hEDS, with the remainder (*n* = 224) having a diagnosis of HSD. This pattern was consistent across the three groups (86.2% of autistic group; 84.9% of high AQ group; 88.8% of non-autistic group; *χ*^2^(4, *N* = 1754) = 6.65, *p* = 0.16, Cramér’s *V* = 0.04). Additionally, the groups did not differ on Beighton score (autistic M = 6.49, SD = 2.95; high AQ M = 6.40, SD = 3.09; non-autistic M = 6.11, SD = 3.29; *H*(2, *N* = 1651) = 1.74, *p* = 0.419).

### Diagnostic experience

Participants reported significant delays between symptom onset and diagnosis—an average of 18.85 years (SD = 12.08). A Kruskal–Wallis *H*-test indicated significant group differences (*H*(2, *N* = 1476) = 6.06, *p* = 0.048); follow-up pairwise comparisons using Dunn’s test with a Bonferroni correction indicated that participants in the non-autistic group had significantly longer delays (mean = 19.40; median = 18.00; *SD* = 12.43) than those in the autistic group (mean = 17.65; median = 15.00; *SD* = 11.63). No significant differences were found between the high AQ group (mean = 18.89; median = 17.00; *SD* = 11.71) and the other two groups.

Most diagnoses were made in rheumatology (74.58%), physiotherapy (5.3%), neurology (1.68%), and gastroenterology (1.29%). The remaining diagnoses were made in ‘other’ primary and secondary health services (*n* = 321; 17.15%). The groups did not significantly differ on the specialism involved in making the diagnosis (*χ*^2^(14, *N* = 1754) = 176.52, *p* = 0.229, Cramer’s *V* = 0.071).

### hEDS/HSD symptom profile

**Fig. 1:**
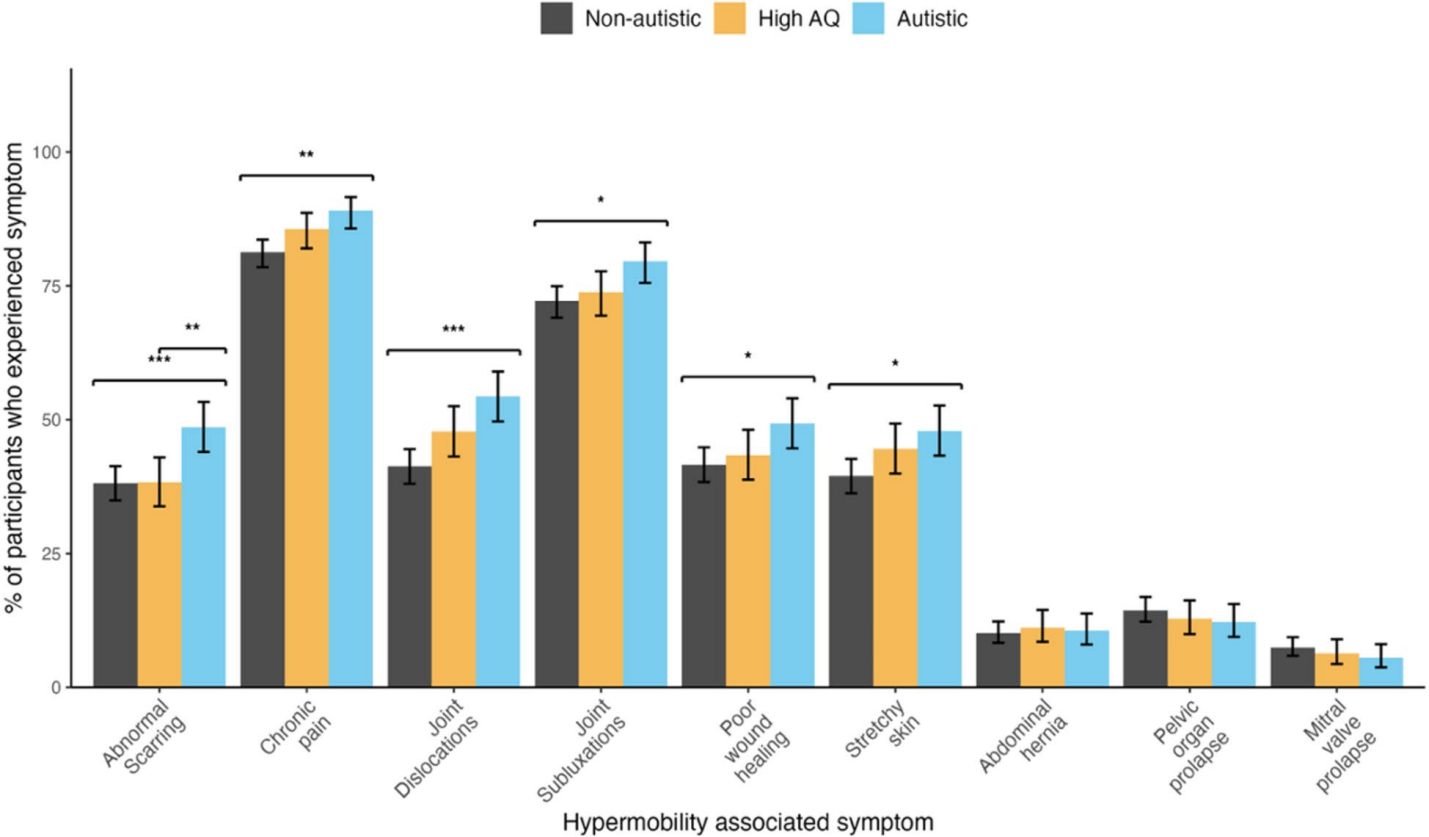
hEDS/HSD-associated symptoms by group; * < 0.05, ** < 0.01, *** < 0.001

Participants were asked if they had ever experienced a range of hEDS/HSD-associated symptoms (Fig. 1, for full details and comparative statistics see Additional file 2, Supplement 2). Participants in the autistic group were significantly more likely to have experienced dermatological, orthopaedic, and pain symptoms compared with the non-autistic group. Generally, the autistic and high AQ groups did not differ, except that more autistic people had experienced abnormal scarring. The high AQ group did not significantly differ from the non-autistic group on any symptom.

### Co-occurring physical and mental health conditions

**Fig. 2:**
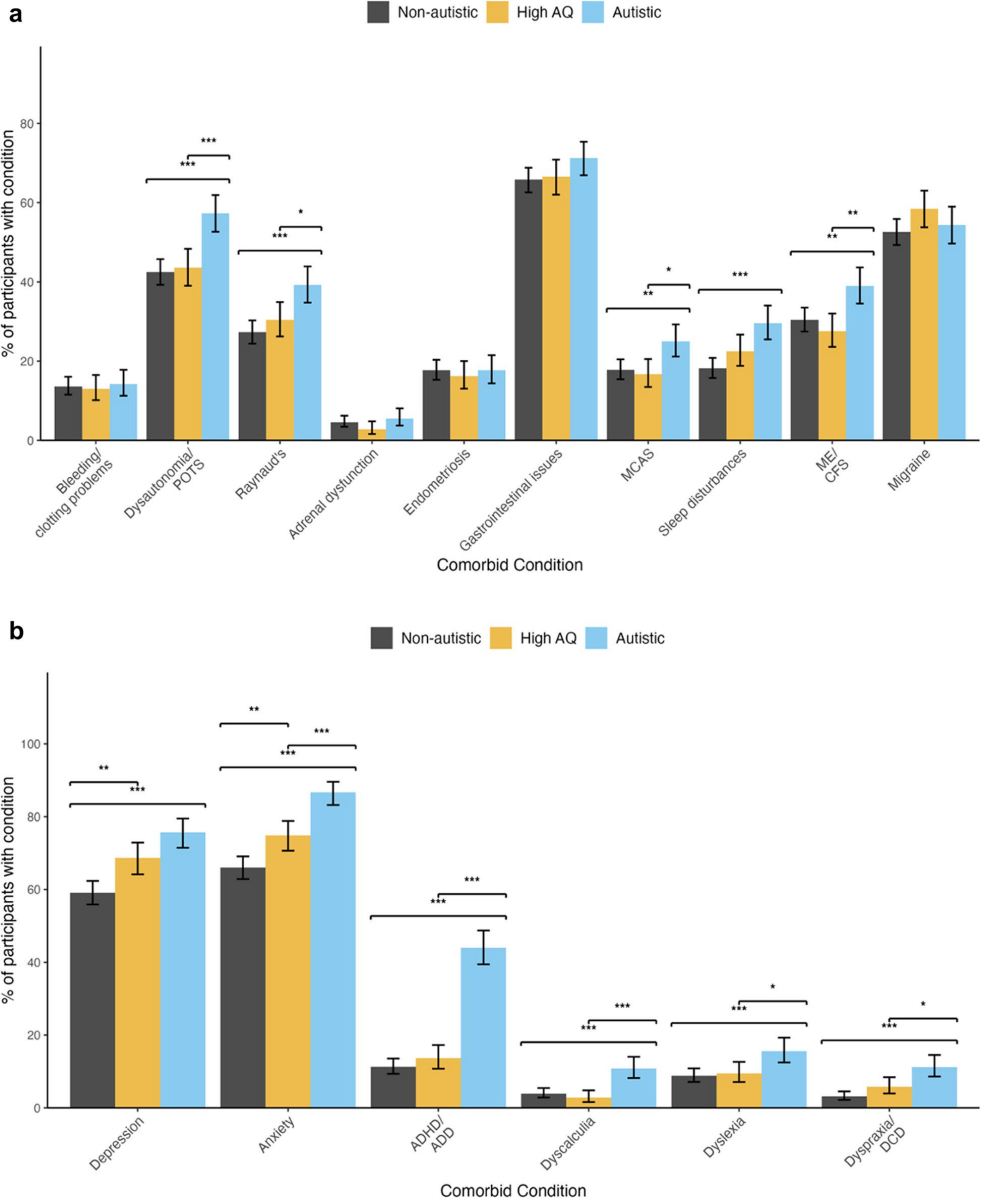
**a** Co-occurring physical health conditions by group; * < 0.05, ** < 0.01, *** < 0.001: MCAS, mast cell activation syndrome; ME/CFS, myalgic encephalomyelitis/chronic fatigue syndrome. **b** Co-occurring mental health conditions and neurodivergence by group; * < 0.05, ** < 0.01, *** < 0.001

Participants were also asked whether they had ever been diagnosed with a range of conditions which commonly co-occur with hEDS/HSD (Fig. 2a; for full statistics see Additional file 2, Supplement 3). Autistic people were more likely to have been diagnosed with co-existing cardiological, neurological, and immune system conditions than those in the non-autistic and high AQ group, as well as sleep disturbances.

Additionally, autistic people were more likely to have been diagnosed with other types of neurodivergence (ADHD, dyspraxia, dyslexia, dyscalculia) and mental health conditions (anxiety, depression) compared with other groups (Fig. 2b). People in the high AQ group were also more likely than those in the non-autistic group to have been diagnosed with anxiety and depression.

### Self-reported mental and physical health

**Fig. 3:**
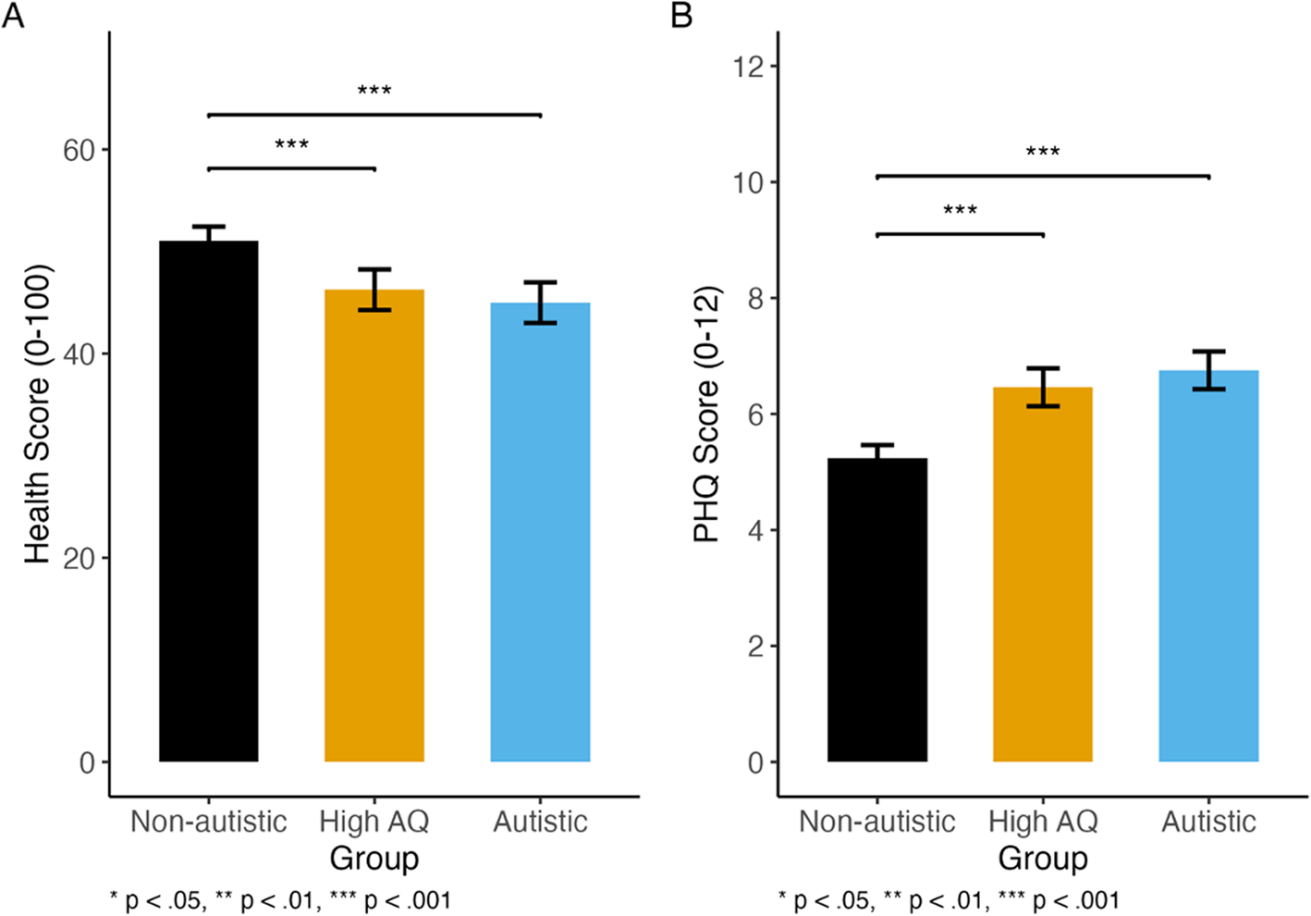
Self-reported physical health score (**A**; out of 100; using EuroQol; higher scores indicate better self-reported physical health) and mental health (**B**; out of 12; using the Patient Health Questionnaire-4 (PHQ-4); higher scores indicate poorer self-reported mental health). Error bars indicate confidence intervals; * < 0.05, ** < 0.01, *** < 0.001

Self-rated mental health was assessed using the PHQ-4 (range: 0–12) with higher scores indicating higher mental distress. The three groups were compared using a linear regression with PHQ score as the dependent variable and group (reference: non-autistic) as the predictor (Fig. 3A). There was a significant difference between the three groups (*F*(2, 1751) = 35.29, *p* < 0.001), with the model accounting for approximately 3.8% of the variance in PHQ-4 scores (adjusted *R*^2^ = 0.04). Pairwise comparisons using Tukey-adjusted contrasts indicated that both the autistic group (mean difference = 1.517, SE = 0.202, *t*(1751) = 7.505, *p* < 0.001, Cohen’s *d* = 0.44, 95% CI [0.32, 0.56]) and high AQ group (mean difference = 1.22, SE = 0.20, *t*(1751) = 6.03, *p* < 0.001, Cohen’s *d* = 0.35, 95% CI [0.24, 0.47]) had significantly higher PHQ-4 scores than the non-autistic group, indicating a higher severity of mental health symptoms, with a moderate effect size. The autistic and high AQ groups did not significantly differ on PHQ scores (autistic mean 6.75, SD = 3.44; high AQ mean = 6.46; SD = 3.38; mean difference = 0.29, SE = 0.24, *t*(1751) = 1.248, *p* = 0.43, Cohen’s *d* = 0.09, 95% CI [− 0.05, 0.22]).

Self-rated physical health was assessed using the EuroQol (EQ-5D-5L; range 0–100) with higher scores indicating better health. The mean health score was 55/100 (see Fig. 3B). A beta regression was performed to examine the effect of autism group (non-autistic, high AQ, autistic) on the health score (health_score_beta; range 0–1). The overall model was statistically significant, with a log-likelihood of 309 on 4 Df and a pseudo-*R*^2^ of 0.02. Estimated marginal means indicated average health scores of 51 for the non-autistic group (95% CI [50, 52]), 46 for the high AQ group (95% CI [44, 48]), and 45 for the autistic group (95% CI [43, 47]). Post hoc comparisons with Bonferroni adjustment showed that the non-autistic group reported significantly higher scores than both the autistic (*p* < 0.001) and high AQ (*p* = 0.0004) groups. The autistic and high AQ groups did not significantly differ (*p* = 1.00).

### Referrals to other clinical services

**Fig. 4:**
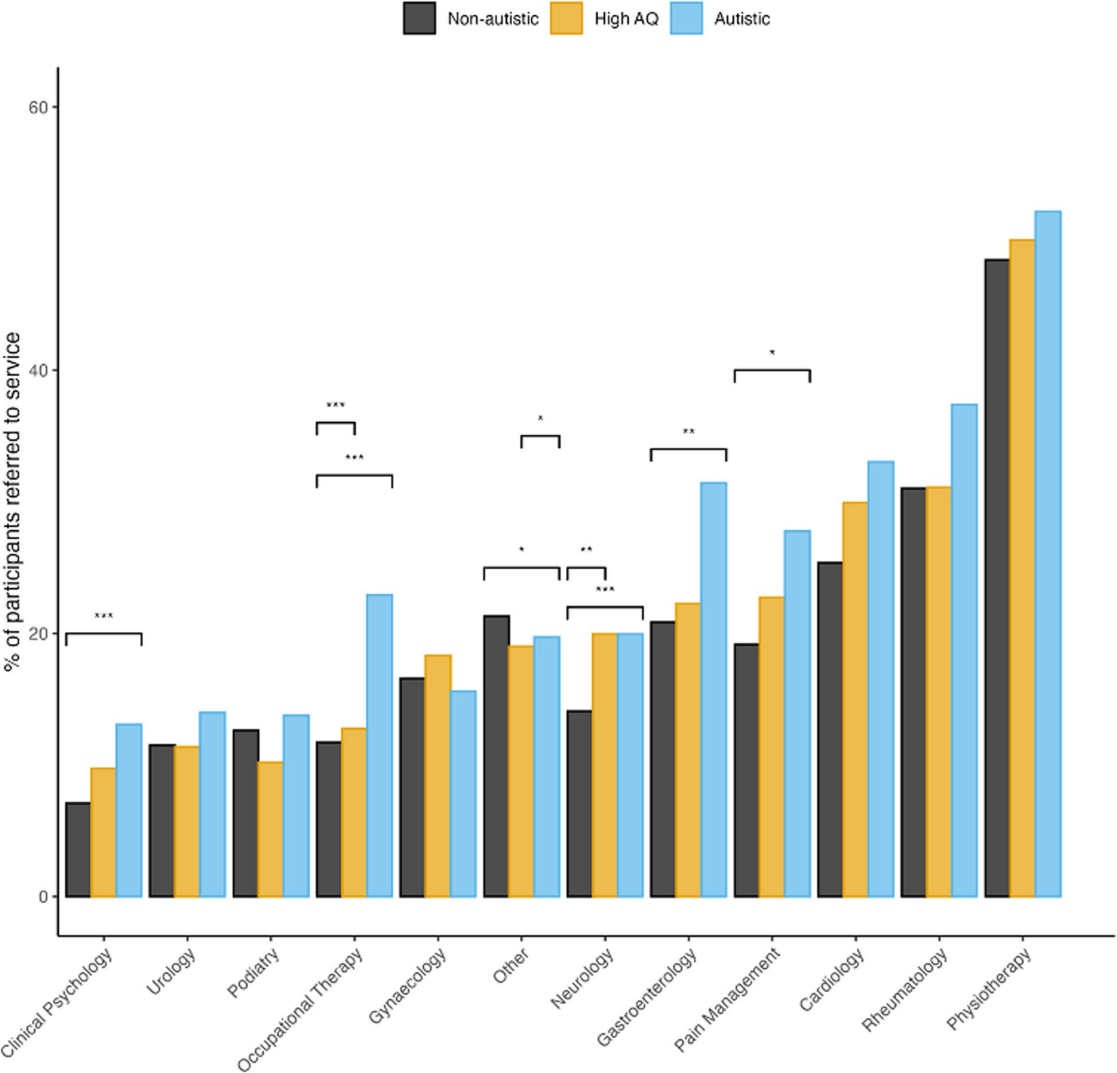
Referrals to other clinical specialities by group; * < 0.05, ** < 0.01, *** < 0.001

Participants were asked which services they had been referred to and accessed within the last 5 years (Fig. 4; for full details, see Additional file 2, Supplement 4). Significantly more autistic respondents had been referred to almost all clinical services than participants in the high AQ or non-autistic group. Compared with non-autistic participants, autistic participants were significantly more likely to have been referred to every service except gynaecology and podiatry: significantly more autistic participants were referred to cardiology (43.7% vs 33.3%, *p* < 0.001), clinical psychology (18.7% vs 9.9%, *p* < 0.001), gastroenterology (36.9% vs 26.9%, *p* < 0.001), neurology (25.0% vs 19.3%, *p* < 0.001), occupational therapy (28.1% vs 17.1%, *p* < 0.001), pain management (32.7% vs 25.0%, *p* < 0.001), physiotherapy (64.5% vs 58.5%, *p* < 0.001), rheumatology (52.8% vs 45.0%, *p* < 0.001), and urology (17.6% vs 13.9%, *p* = 0.01). Compared with high AQ participants, significantly more autistic participants had been seen in cardiology (43.7% vs 36.0%, *p* < 0.001), clinical psychology (18.7% vs 12.6%, *p* < 0.001), gastroenterology (36.9% vs 28.6%, *p* < 0.001), occupational therapy (28.1% vs 20.3%, *p* < 0.001), podiatry (20.3% vs 16.4%, *p* = 0.03), rheumatology (52.8% vs 46.4%, *p* < 0.001), and urology (17.6% vs 13.8%, *p* = 0.02).

Participants in the high AQ group were also significantly more likely to have seen a clinical psychologist (12.6% vs 9.9%, *p* = 0.03), neurologist (23.4% vs 19.3%, *p* = 0.01), occupational therapist (20.3% vs 17.1%, *p* = 0.04), pain management specialist (29.2% vs 25.0%, *p* = 0.01), and physiotherapist (62.6% vs 58.5%, *p* = 0.04), compared with those in the non-autistic group.

### Non-clinical support

The groups differed on whether they received support from a carer (Pearson’s chi-square *χ*^2^(2, *N* = 1754) = 54.69, *p* < 0.001). Post hoc pairwise comparisons with Bonferroni corrections indicated that respondents in the autistic (39.3%) and high AQ (32.7%) groups were more likely to have a carer compared with those in the non-autistic group (20.8%, *p* < 0.001).

The groups also differed on whether they accessed support from charities (*χ*^2^(4, *N* = 1754) = 11.53, *p* = 0.021). Post hoc pairwise comparisons using Bonferroni corrections indicated that autistic participants (17.4%) were significantly more likely to report accessing charity support than those with high AQ scores (10.0%; *p* = 0.011). The non-autistic group (15.1%) did not significantly differ from either the autistic (*p* = 1.00) or non-autistic group (*p* = 0.086).

### Employment and benefits

Overall, 54% of participants were in full- or part-time employment, and 46% were unemployed. There were significant differences between the groups (Pearson chi-square test *χ*^2^(2, *N* = 1754) = 23.26, *p* < 0.001), with post hoc comparisons indicating that autistic people were significantly less likely to be employed (44.7%) than both the high AQ group (56.4%; *p* = 0.002, Cramér’s *V* = 0.12) and the non-autistic group (58.6%; *p* < 0.001, Cramér’s *V* = 0.13). The difference in employment rates between the high AQ and non-autistic groups was not statistically significant (*p* = 1.00).

The groups also differed on whether they received disability benefits (*χ*^2^(2, *N* = 1754) = 45.86, *p* < 0.001), with post hoc pairwise comparisons with a Bonferroni correction indicating that more people in the autistic group (61.3%) were on benefits compared to both the high AQ group (48.8%; *p* = 0.001, Cramér’s *V* = 0.13) and the non-autistic group (41.5%; *p* < 0.001, Cramér’s *V* = 0.19). Furthermore, there were more people in the high AQ group claiming benefits than the non-autistic group (*p* = 0.035, Cramér’s *V* = 0.07).

### Predictors of self-reported physical and mental health

#### Missing data and variable selection

Initial data screening identified a total of 2.4% missing data in the entire dataset. Predictors with substantial missing data (> 10%), primarily resulted from conditional survey items not administered to all participants in the online survey system. We thus excluded variables ‘dyspraxia/DCD (39%)’, ‘dyslexia (39%)’, and ‘dyscalculia’ (39% missing), years since diagnosis (15%), delay in diagnosis (16%), and being diagnosed with a subtype of EDS (13%).

Subsequently, the dataset was split into subsets for the self-reported physical health (from the EuroQol; *n* = 1626) and mental health (from the PHQ; *n* = 1754) analyses. The remaining missing data (< 0.1%), presumed missing at random, were imputed using Multiple Imputation by Chained Equations [66] with predictive mean matching, which maintains statistical power, preserves observed distributions, and accommodates non-linear associations without stringent assumptions [31]. We generated five imputed datasets (*m* = 5) using predictive mean matching (‘pmm’) as the imputation method. The process was run for ten iterations (maxit = 10) for each imputed dataset. To assess the stability of the imputation process, we visually inspected plots of the regression coefficients from the models fitted to each of the five imputed datasets to ensure there were no substantive differences between them. The imputation model included all predictors, and final model estimates were pooled across the five datasets according to Rubin’s rules.

Given our research aims, we used participant group (autistic, non-autistic, or high AQ) and explicitly excluded autism diagnosis to avoid redundancy. Additionally, for the PHQ-4 analysis, diagnosis of anxiety and diagnosis of depression were excluded to avoid redundancy. Comorbidities present in less than 1% of the sample (porphyria, alpha-1 antitrypsin deficiency, and tethered cord syndrome) were removed.

In these analyses, we aimed to examine which variables predict self-reported physical and mental health. As there were numerous potential predictors (*n* = 50), a more manageable set of predictors was identified using *glmnet* [26]. This works by applying a penalty that shrinks the regression coefficients of all predictors towards zero. Crucially, it can force the coefficients of the least influential variables to become exactly zero, effectively removing them from the model and leaving a more parsimonious set of key predictors. We considered two candidate models based on cross-validation results: one using the lambda value that minimised prediction error (*λ*ₘᵢₙ) and another using the lambda within one standard error of the minimum (*λ*_1_ₛₑ), which offers a more parsimonious set of predictors. Here, we will present the most parsimonious models, though *λ*ₘᵢₙ models are available in Additional file 2, Supplement 8 and 9.

#### Predictors of self-reported physical health

Model comparison (see Additional file 2, Supplement 8A for full details) suggested that a beta-regression model was the best fitting. The model accounted for 21% of the variance (pseudo-*R*^2^ = 0.21) and exhibited excellent precision (*φ* = 2 990, *z* = 28.44, *p* < 0.001), with quantile‐residual diagnostics showing no systematic departures from uniformity (range: 3.4 to 3.8; median ≈ 0). Model diagnostics and sensitivity checks (e.g. zero/one-inflation tests, heteroskedasticity checks, influence analyses) provided no evidence of misfit, supporting confidence in these findings (see Additional file 2, Supplement 6 for full details).

**Fig. 5:**
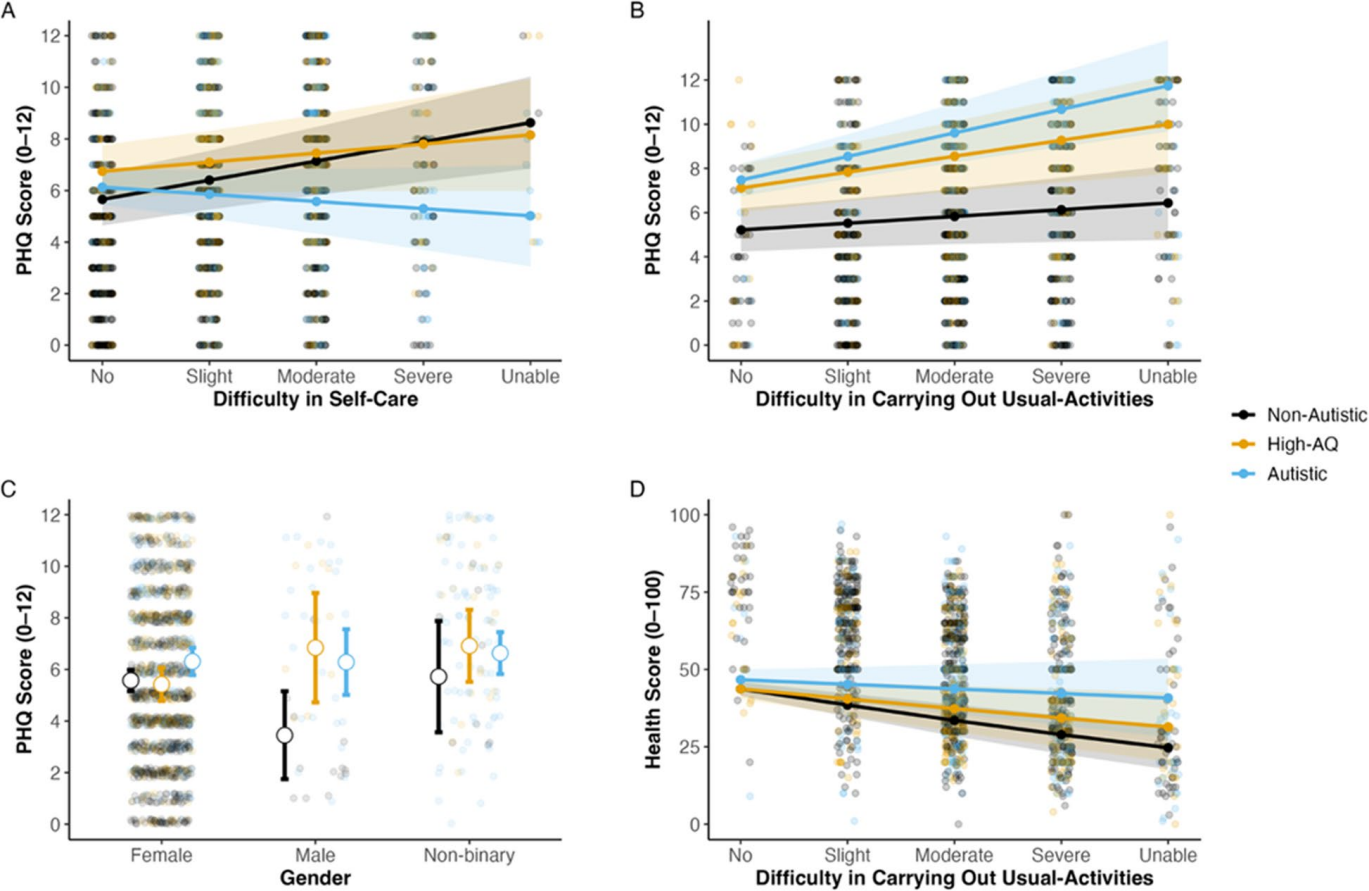
Estimated marginal means showing the modelled two-way interactions from the PHQ-4 and health score models. **A** The effect of difficulty in carrying out self-care on PHQ-4 scores. **B** The effect of difficulty in carrying out usual activities on PHQ-4 scores. **C** The differences in PHQ-4 scores by gender. **D** The effect of difficulty in carrying out usual activities on health scores. Solid lines represent the estimated regression slopes and shaded bands represent the corresponding 95% confidence intervals derived from simple-slope analysis. Points represent the estimated marginal means at specific predictor values

Lower health scores were predicted by limitations in usual activities (*B* = − 0.22, SE = 0.04, 95% CI [− 0.29, − 0.14], *z* = − 5.62, *p* < 0.001, corresponding to a 5.5-point decrease per SD); mobility problems (*B* = − 0.11, SE = 0.04, 95% CI [− 0.18, − 0.03], *z* = − 2.84, *p* = 0.005, corresponding to a 2.3-point decrease per SD); and anxiety and depression (*B* = − 0.10, SE = 0.03, 95% CI [− 0.15, − 0.04], *z* = − 3.61, *p* < 0.001, corresponding to a 2.1-point decrease per SD). Additionally, limitations in usual activities significantly interacted with group (*B* = 0.16, SE = 0.07, 95% CI [0.03, 0.28], *z* = 2.38, *p* = 0.017), with simple-slope tests (Tukey-adjusted) showing a 1-SD increase in difficulty carrying out one’s usual activities predicted a 5.4-point decrease in health scores for those in the non-autistic group but a 1.5-point decrease in health scores for those in the autistic group (see Fig. 5). The difference between slopes was 3.9 points (SE = 0.02, *z* = 2.385, *p* = 0.045). This indicates that while limitations to usual activities are generally detrimental to physical health, this is a particularly strong negative impact for non-autistic people.

#### Predictors of self-reported mental health

A multiple linear regression was conducted with PHQ-4 scores (range 1–12) as the dependent variable. The most parsimonious model retained eight predictors (see Additional file 2, Supplement 9 for full details), including age and gender; a diagnosis of ADHD, whether a GP had initiated care management steps relating to hEDS/HSD, and access to an hEDS/HSD-aware medical professional. The model also included three functional and quality of life domains from the EQ-5D-5L questionnaire (numeric variables scaled from 1 to 5), including self-care (assessing problems with washing or dressing), usual activities (assessing problems with work, study, or leisure), and pain/discomfort, with higher scores indicating greater impairment across all three domains. The model was statistically significant, *F*(26, 1727) = 11.79, *p* < 0.001, and explained approximately 14% of the variance (adjusted *R*^2^ = 0.138).

Several predictors emerged as significant main effects. Participants who were older (*b* = − 0.53, SE = 0.09, *t*(1727) = − 5.87, *p* < 0.001, 95% CI [− 0.70, − 0.35]) and female (*b* = − 2.12, SE = 0.86, *t*(1727) = − 2.48, *p* = 0.013, 95% CI [− 3.80, − 0.44]) reported lower PHQ-4 scores, indicating better mental health. Additionally, participants who reported that their general practitioner had initiated additional treatment steps had significantly lower PHQ-4 scores (*b* = − 0.64, SE = 0.24, *t*(1727) = − 2.66, *p* = 0.008, 95% CI [− 1.10, − 0.17]).

Conversely, those who experienced difficulty with self-care (*b* = 0.79, SE = 0.16, *t*(1727) = 4.94, *p* < 0.001, 95% CI [0.48, 1.11]) and had difficulty with performing usual activities (*b* = 0.32, SE = 0.16, *t*(1727) = 2.08, *p* = 0.038, 95% CI [0.02, 0.63]) had higher PHQ-4 scores indicating poorer mental health.

Furthermore, three significant interaction effects were identified. Firstly, a significant interaction between group (autistic vs non-autistic reference) and self-care difficulty emerged (*b* = − 1.09, SE = 0.27, *t*(1727) = − 4.04, *p* < 0.001, 95% CI [− 1.61, − 0.57]), indicating that while greater self-care difficulties were associated with higher PHQ-4 scores overall, that increased difficulty with self-care had a stronger impact on the mental health of non-autistic participants compared with autistic participants.

Secondly, an interaction between group and difficulties with usual activities was found (*b* = 0.81, SE = 0.27, *t*(1727) = 3.00, *p* = 0.003, 95% CI [0.29, 1.33]), with a stronger positive association between functional limitations and higher PHQ-4 scores in the autistic group compared with the non-autistic group. This indicates that difficulty with usual activities had a greater negative impact on autistic people’s mental health compared with non-autistic people.

Finally, a significant interaction between group (high AQ vs non-autistic reference) and gender was identified (*b* = 3.54, SE = 1.36, *t*(1727) = 2.60, *p* = 0.009, 95% CI [0.88, 6.21]). This indicates that the differences in PHQ-4 scores between the genders within the high AQ group were significantly larger than the non-autistic group. Probing this interaction, results indicated that females had substantially higher PHQ-4 scores in the high AQ group, indicating that high AQ females experience more symptoms of anxiety and depression in relation to other genders within the high AQ group, and females in other diagnostic groups.

## Discussion

In this study, we aimed to examine whether autistic people, people with high levels of autistic traits, and people with low levels of autistic traits experience and are impacted by hEDS/HSD in similar or different ways. Additionally, we explored what predicts self-reported physical and mental health outcomes in these groups. Ongoing research by Ritelli et al. [53] is examining potential biomarkers of hypermobility, which may enhance diagnosis and deepen our understanding of the link between hypermobility and autism. This research could potentially inform precision medicine treatments for autistic people with symptomatic hypermobility. However, it will take time for any potential underlying biological mechanism to be realised. Presently, converging lines of evidence suggest a significant link between the two, and as such, it is important to improve our understanding of the experiences of people with both hEDS/HSD and autism. Understanding the experiences of these groups and the barriers they face to health and social care provision is crucial to minimise negative health and wellbeing outcomes.

Our survey generated a high number of responses. Around 25% of respondents were autistic, and a further 25% had high AQ scores—proportions that are in line with previous studies [3]. All participants reported significant delays in receiving a diagnosis of hEDS/HSD with an average of almost 19 years between first symptoms and diagnosis. This is not unusual given the ‘diagnostic odyssey’ often experienced by hEDS/HSD patients [29, 30]. Perhaps unexpectedly given the difficulties autistic people face in accessing healthcare [69], participants in the non-autistic group had the longest delay to diagnosis.

The three groups did not differ on Beighton scores which assesses joint hypermobility. However, autistic people had been diagnosed with more hEDS/HSD-associated symptoms than non-autistic people across multiple symptom categories. This suggests that Beighton score may not capture group differences in hypermobility severity in this sample. Interestingly, the high AQ and non-autistic groups did not significantly differ on their experiences of hEDS/HSD symptoms.

Autistic respondents were more likely to be diagnosed with a range of neurodivergent conditions, including ADHD, dyscalculia, dyslexia, and dyspraxia. This is unsurprising given the high levels of co-occurrence of neurodivergences: it is estimated that between 30 and 50% of autistic people have symptoms of ADHD [18, 40], and one recent study finding that over 70% of children referred for an autism diagnosis scored greater than the median for one additional neurotype, indicating an elevated level of other neurodivergent traits [39].

Autistic people in this study were also more likely to experience anxiety and depression. Again, this is perhaps unsurprising given the high prevalence of these conditions within the autistic population [33]. Anxiety and depression were also more common in the high AQ group compared with the non-autistic group, aligning with previous research linking autistic traits with depressive and anxious symptomatology [54]. However, there were no differences between the high AQ group and the non-autistic group on physical health conditions or neurodivergence. This indicates that while high AQ people might experience higher rates of anxiety and depression, subclinical autistic traits do not appear to be associated with the same physical health profile as those with a clinical diagnosis of autism. It is important to note that high AQ participants were also more likely to have been referred to other clinical specialities including psychology, occupational therapy, pain management, neurology, and physiotherapy, compared with non-autistic people. They were also more likely to have support from a carer and be in receipt of benefits. Thus, future research may not only focus on a binary of ‘autistic’ and ‘non-autistic’ people with hEDS/HSD, but consider those with high autistic traits and their specific hypermobile support needs.

Autistic respondents were also more likely to have a diagnosis of several co-occurring physical health conditions, including dysautonomia/POTS, chronic fatigue/ME, and mast cell activation syndrome. Unsurprisingly, autistic respondents who rated their physical and mental health as poorer than non-autistic and high AQ respondents were more likely to have been referred to clinical services across a range of specialities. While there are elevated rates of many health conditions for autistic people [71, 72], identifying the specific conditions that commonly occur with hEDS/HSD for autistic people may inform their care and support and help address diagnostic overshadowing [36]. It is also important for future research to examine whether there is an underlying ‘autistic hypermobile endotype’, with specific biological mechanisms responsible for autistic people experiencing elevated symptomatic hypermobility alongside increased rates of co-occurring physical and mental health conditions, or whether this is simply due to the fact that autistic people are more likely to experience a myriad of health conditions, and these are coincidentally occurring in autistic people with symptomatic hypermobility [33, 34, 50, 57, 68].

When examining which factors predicted self-reported physical health outcomes, greater impairments in mobility, anxiety, and depression were associated with lower perceived physical health. While participants were also generally negatively impacted by limitations to usual activities, this was particularly so for non-autistic participants. However, the opposite was found when considering self-rated mental health, where autistic peoples’ mental health was more negatively impacted by being unable to do their usual activities than non-autistic people. This may be because routines can be particularly important for autistic people and have been shown to be a protective factor for mental health [32]. Finally, women in the high AQ group were more likely to rate their mental health as poorer compared with other genders in that group. While this may need to be interpreted with caution given the high number of females in this group, this aligns with research indicating that females with high autistic traits or undiagnosed autism experience significant symptoms of poor mental health [6]. If some of these women are indeed autistic, screening for autism following a diagnosis of hEDS/HSD may be warranted [15].

There are some limitations to this research. Firstly, though the sample was very large and thus hopefully representative, it was nonetheless a self-selecting sample recruited from the EDS UK website and via their social media. It may have reached a specific type of participant who is engaged with or aware of a condition-specific charity, and thus their condition may not be as well-managed as others with hEDS/HSD who are not engaged with this charity. Secondly, participants were mostly white, and we do not know if participants from racialised minorities experience similar or different experiences of hEDS/HSD with or without autism, or additional barriers to healthcare for hEDS/HSD. Thirdly, participants were mostly female, though this fits with previous research on hypermobility [60] and may be due to symptomatic hypermobility being associated with sex hormones [27]. Additionally, we did not ask whether participants had a co-existing intellectual disability, though the method of data collection is likely to have excluded some participants with intellectual disability. Autistic people with intellectual disability are generally under-represented in research, including self-reported assessment studies such as this, and future work that includes autistic people with additional needs is warranted. Finally, autistic traits were assessed using the AQ, which is a very frequently used screening measure for autistic traits [55]. However, this measure has been criticised as it may not capture autistic traits equally across genders [38], does not account for cultural differences in the expression of social and communication behaviours [24], and is built around a fixed set of behaviours, traits, and perceived deficits considered typical of autism which may not represent the diversity of the autistic community [35]. This measure was selected as it is commonly used in autism research; however, its limitations should be recognised particularly in relation to gender, given the large number of female participants in this study.

Future work may focus on the integration of both screening for autism after a hEDS/HSD diagnosis and screening for hEDS/HSD after an autism diagnosis. As both conditions tend to be diagnosed by professionals in different clinical fields, their co-occurrence can often be overlooked [11, 12], and for both conditions, diagnostic overshadowing is common [28]. Joint laxity may be assumed to be related to autism rather than a connective tissue disorder and so is often overlooked,and hEDS/HSD are overwhelmingly diagnosed in females, and so autism is often overlooked given [11, 12]. Integrating screening for these commonly co-occurring conditions may reduce the very long time taken to obtain diagnosis, and allow access to support and care much sooner.

Emerging work is also focusing on the relationship between hypermobility syndromes and other types of neurodivergence such as ADHD [4, 37], particularly as people with ADHD also have difficulties accessing healthcare [25]. Given the high co-occurrence of autism and ADHD [18, 40], future work may focus on disentangling what underlies this relationship.

Future research may also consider exploring recommendations for autistic-friendly healthcare, with a particular focus on conditions such as hEDS/HSD where there is a significant overlap. Currently, autistic people face significant barriers to accessing healthcare [69] and poorer health outcomes [21] than non-autistic people, and focusing support in these areas may have significant benefits particularly for chronic, lifelong health conditions such as hEDS/HSD.

## Conclusions

This study indicates that autistic and non-autistic people may experience hEDS/HSD in different ways. This may, in turn, affect the type of supports that are most beneficial to them. While interventions exist to support healthcare experience of autistic people (see [7] & [70] for reviews), the authors have not found any interventions specifically relating to symptomatic hypermobility health care for autistic people. Given the high co-occurrence of autism and hEDS/HSD, and that fact that these are lifelong conditions often requiring multidisciplinary care and support, it is important to examine and explore the autistic experience of hypermobility, care, and outcomes, and which interventions and supports may be most beneficial.

## Supplementary Information

Additional file 1: The full survey completed by participants.

Additional file 2: Additional statistical detail, model comparison, and figures supplemental to the main text.

## Data Availability

The datasets generated and/or analysed during the current study are not publicly available due to participants not providing consent for their data to be openly available, but are available from the corresponding author on reasonable request.

## Abbreviations

AQ: Autism Spectrum Quotient
hEDS: Hypermobile Ehlers-Danlos syndrome
HSD: Hypermobility spectrum disorder
PHQ-4: Patient Health Questionnaire-4
