# Supplementary material for: Health experiences and outcomes of autistic and non-autistic adults with hypermobile Ehlers-Danlos syndrome and hypermobility spectrum disorder
Source: BMC Med. 2026 Feb 25;24:193. doi: 10.1186/s12916-026-04713-2 (PMC13040810; doi:10.1186/s12916-026-04713-2)
Supplement: Supplementary file 1 — Additional file 1: The full survey completed by participants. [file 12916_2026_4713_MOESM1_ESM.pdf]

# hEDS-START 1 - Understanding the lived experience of patients with hypermobile Ehlers-Danlos Syndrome and Hypermobile Spectrum Disorder

Online Survey and Consent Form

About this study

The purpose of this study is to find out more about the lived experience of hypermobile Ehlers-Danlos Syndrome (hEDS) or Hypermobile Spectrum Disorder (HSD). You are invited to take part in the survey if you have previously been diagnosed with hEDS/HSD.

A detailed information sheet can be found here.

This research is a survey that will collect information about your lived experience with hEDS/HSD, its impact on your quality of life, other diagnoses you may have, and your experience of the healthcare system. This survey should take around 30 minutes of your time. The survey is completely anonymous and there is no obligation to provide any personal information.

At the end of the survey, you will be asked if you would like to leave your contact details so that the research team can contact you with information about future ethically approved research projects. Please note that if you take part in hEDS-START 1 there is no obligation whatsover to take part in future research.

Who can take part in this study?

This survey is designed to be completed by anyone over the age of 16 with a diagnosis of hEDS/HSD.

What about privacy and confidentiality?

Responses to the survey will be anonymous and will be stored securely on a password protected computer at the University of Edinburgh for 15 years. After this time, the data will be destroyed. Only the researchers conducting this survey and the study coordinator will have access to the data. The results of the survey may be published but will not include identifiable information.

Can I obtain a summary of survey findings?

At the end of the survey, you will have the option to download a PDF copy of your survey answers.

What if I have any questions?

If you have any questions about the survey you can contact Kathryn.Berg@ed.ac.uk or phone 0131 651 8755.

What do I do now if I wish to take part?

Please read the detailed information sheet and take enough time to ensure you know what is involved. If you have read and understood the above information and would like to participate in the survey, please click on the following boxes to indicate that you consent to take part in the survey. If you do not want to take part please close the web page on your browser

Please complete the survey below.

Thank you!

## Consent

| | |
|---|---|
| I confirm that I have read and understand the information sheet (29-11-2023 Version 2.0) for the above study. | ◯ Yes<br>◯ No |
| I have had the opportunity to consider the information, ask questions, and have had these questions answered satisfactorily. | ◯ Yes<br>◯ No |
| I understand that relevant sections of the data collected during the study will be looked at by individuals from the Sponsor (University of Edinburgh). I give permission for these individuals to have access to my data. | ◯ Yes<br>◯ No |
| I understand that data collected about me during the study may be converted to anonymised data. | ◯ Yes<br>◯ No |
| I agree to take part in the above study. | ◯ Yes<br>◯ No |

**Eligibility Criteria This survey is designed to be completed only by people who are aged 16 or older. We kindly request that you do not complete this survey if you are under 16 years of age.**

| | |
|---|---|
| Are you aged 16 or older? | ◯ Yes<br>◯ No |
| Do you have a diagnosis of hypermobile Ehlers-Danlos Syndrome or Hypermobile Spectrum Disorder? | ◯ Yes<br>◯ No |

## Demographics

| | |
|---|---|
| What country do you currently reside in? | ◯ Scotland<br>◯ England<br>◯ Northern Ireland<br>◯ Wales |
| What is your age range? | ◯ 16 to 24<br>◯ 25 to 34<br>◯ 35 to 44<br>◯ 45 to 54<br>◯ 55 to 64<br>◯ 65 to 74<br>◯ 75 or older |
| What sex were you assigned at birth? | ◯ Female<br>◯ Male<br>◯ Other |
| What is the gender with which you identify? | ◯ Female<br>◯ Male<br>◯ Non-Binary |
| What is your ethnicity? | ◯ White English, Welsh, Scottish, Northern Irish or British<br>◯ White Irish<br>◯ Gypsy or Irish Traveller<br>◯ Roma<br>◯ Any other White background<br>◯ White and Black Caribbean<br>◯ White and Black African<br>◯ White and Asian<br>◯ Any other mixed background<br>◯ Indian<br>◯ Pakistani<br>◯ Bangladeshi<br>◯ Chinese<br>◯ Any other Asian background<br>◯ Caribbean<br>◯ African<br>◯ Any other Black, Black British, or Caribbean background<br>◯ Arab<br>◯ Any other ethnic group |

## Diagnosis

| Question | Response |
| --- | --- |
| Do you have a confirmed diagnosis of hEDS? This includes previous terminology such as EDS hypermobility type and EDS Type III. | ◯ Yes<br>◯ No |
| If no, what is your diagnosis? | ◯ Hypermobility Spectrum Disorders<br>◯ Symptomatic Hypermobility<br>◯ Benign Joint Hypermobility Syndrome<br>◯ Other |
| Which health care professional were you diagnosed by? | ◯ Doctor<br>◯ Nurse<br>◯ Occupational Therapist<br>◯ Physiotherapist<br>◯ Podiatrist<br>◯ Dentist<br>◯ Other |
| In which specialty were you diagnosed? | ◯ Rheumatology<br>◯ Cardiology<br>◯ Gastroenterology<br>◯ Urology<br>◯ Gynaecology<br>◯ Neurology<br>◯ Dentistry<br>◯ Other |
| If other, please elaborate: | __________________________________ |
| Were you diagnosed by a healthcare professional in the NHS or privately? | ◯ NHS<br>◯ Private |
| Where were you officially diagnosed? | ◯ Scotland<br>◯ England (London)<br>◯ England (Other)<br>◯ Northern Ireland<br>◯ Wales<br>◯ Other European country<br>◯ Other international country |
| Which year were you diagnosed? | __________________________________ |
| How many years were there between the presentation of your first symptoms and an official diagnosis by a healthcare professional? | __________________________________ |
| Have you been diagnosed with any other subtype of EDS? | ◯ Yes<br>◯ No |

Please confirm which other subtype of EDS you have been diagnosed with:

- ☐ Classical EDS (cEDS)
- ☐ Classical-like EDS (clEDS)
- ☐ Cardiac-valvular EDS (cvEDS)
- ☐ Vascular EDS (vEDS)
- ☐ Arthrochalasia EDS (aEDS)
- ☐ Dermatosparaxis EDS (dEDS)
- ☐ Kyphoscoliotic EDS (kEDS)
- ☐ Brittle Cornea Syndrome (BCS)
- ☐ Spondylodysplastic EDS (spEDS)
- ☐ Musculocontractural EDS (mcEDS)
- ☐ Myopathic EDS (mEDS)
- ☐ Periodontal EDS (pEDS)

## Clinical Features

What is your Beighton score?
- ◯ 1
- ◯ 2
- ◯ 3
- ◯ 4
- ◯ 5
- ◯ 6
- ◯ 7
- ◯ 8
- ◯ 9
- ◯ Unknown

Do you have family members with a clinical diagnosis of, or suspected hEDS or HSD
- ◯ Yes
- ◯ No

If yes, please state:
- ☐ Mother ☐ Father ☐ Sibling
- ☐ Cousin ☐ Aunt/Uncle
- ☐ Grandparent ☐ Child
- ☐ Grandchild ☐ Niece/Nephew
- ☐ Other

If other, please elaborate: ______________________

Have you ever been diagnosed with any of the following conditions?

- ☐ Joint Dislocations
- ☐ Joint Subluxations
- ☐ Dysautonomia/Postural Orthostatic Tachycardia Syndrome (POTS)
- ☐ Mast Cell Activation Syndrome (MCAS)
- ☐ Gastrointestinal manifestations (e.g. gastroparesis, IBS, GORD)
- ☐ Autism spectrum disorder (ASD)
- ☐ Attention Deficit Disorder (ADD)
- ☐ Dyspraxia/DCD
- ☐ Dyslexia
- ☐ Dyscalculia
- ☐ Migraine
- ☐ Cranio-cervical instability (CCI)/Atlanto-axial instability (AAI)
- ☐ Chiari malformation
- ☐ Tethered cord syndrome
- ☐ Sleep disturbances (e.g. sleep apnea, narcolepsy)
- ☐ Anxiety
- ☐ Depression
- ☐ Mitral valve prolapse/regurgitation
- ☐ Other heart valve issues (tricuspid, aortic, pulmonic)
- ☐ Poor wound healing
- ☐ Abnormally stretchy skin
- ☐ Abnormal Scarring
- ☐ Myalgic encephalomyelitis/chronic fatigue syndrome (ME/CFS)
- ☐ Bleeding or clotting problems
- ☐ Adrenal dysfunction
- ☐ Chronic pain
- ☐ Abdominal hernia(s)
- ☐ Pelvic organ prolapse
- ☐ Porphyria
- ☐ Alpha-1 antitrypsin deficiency
- ☐ Raynaud's phenomenon
- ☐ Endometriosis
- ☐ Other

If other, please elaborate:

__________________________________

Have you had an echocardiogram to evaluate heart valve function?

- ◯ Yes
- ◯ No

**Your Experiences of the Healthcare System Please answer the following questions with specific reference to your hEDS diagnosis.**

| | |
|---|---|
| Has your GP initiated steps to manage your care? | ◯ Yes<br>◯ No |
| Has a medical professional suggested counselling or support groups to help you manage your mental health? | ◯ Yes<br>◯ No |
| Have you been referred to a physiotherapist as part of your clinical care? | ◯ Yes, NHS Referral<br>◯ Yes, Private Referral<br>◯ No |
| Have you been referred to an occupational therapist as part of your clinical care? | ◯ Yes, NHS Referral<br>◯ Yes, Private Referral<br>◯ No |
| Have you been referred to a podiatrist as part of your clinical care? | ◯ Yes, NHS Referral<br>◯ Yes, Private Referral<br>◯ No |
| Have you been referred to a pain management course as part of your clinical care? | ◯ Yes, NHS Referral<br>◯ Yes, Private Referral<br>◯ No |
| Have you been referred to a pain management team as part of your clinical care? | ◯ Yes, NHS Referral<br>◯ Yes, Private Referral<br>◯ No |
| Have you been referred to a clinical psychologist as part of your clinical care? | ◯ Yes, NHS Referral<br>◯ Yes, Private Referral<br>◯ No |
| Have you been referred to a residential programme as part of your clinical care? | ◯ Yes, NHS Referral<br>◯ Yes, Private Referral<br>◯ No |
| Do you use any equipment/supports on a regular basis? | ◯ Yes, provided by the NHS<br>◯ Yes, I bought these myself<br>◯ No |
| How often do you see a medical professional regarding your condition? | ◯ I am currently in hospital<br>◯ More than once per week<br>◯ Once weekly<br>◯ Fortnightly<br>◯ Monthly<br>◯ Every two months<br>◯ Every 6 months<br>◯ Yearly<br>◯ Only if required<br>◯ I prefer not to see medical professionals |

| | |
|---|---|
| Which services have you accessed in the last year as a result of your diagnosis? | ☐ Rheumatology<br>☐ Cardiology<br>☐ Urology<br>☐ Gastroenterology<br>☐ Gynaecology<br>☐ Neurology<br>☐ Podiatry<br>☐ Physiotherapy<br>☐ Occupational Therapy<br>☐ Clinical Psychology<br>☐ Pain Management<br>☐ Other |
| Which services have you accessed in the last 3 years as a result of your diagnosis? | ☐ Rheumatology<br>☐ Cardiology<br>☐ Urology<br>☐ Gastroenterology<br>☐ Gynaecology<br>☐ Neurology<br>☐ Podiatry<br>☐ Physiotherapy<br>☐ Occupational Therapy<br>☐ Clinical Psychology<br>☐ Pain Management<br>☐ Other |
| Which services have you accessed in the last 5 years as a result of your diagnosis? | ☐ Rheumatology<br>☐ Cardiology<br>☐ Urology<br>☐ Gastroenterology<br>☐ Gynaecology<br>☐ Neurology<br>☐ Podiatry<br>☐ Physiotherapy<br>☐ Occupational Therapy<br>☐ Clinical Psychology<br>☐ Pain Management<br>☐ Other |
| Do you have an HSD/hEDS-aware medical professional that you can go to in emergencies and for questions? | ◯ Yes, NHS<br>◯ Yes, privately<br>◯ No |
| If yes, please elaborate: | |
| Have you ever accessed HSD/hEDS-related medical care outside of Scotland? | ◯ Yes<br>◯ No |
| If yes, please elaborate: | |

## Psychosocial Impact of Diagnosis

Do you currently work?

◯ Yes ◯ No

If yes, please indicate:

◯ Full-time ◯ Part-time

If no, did you have to leave employment because of your diagnosis?

◯ Yes ◯ No

Are you currently on disability benefits as a result of your diagnosis?

◯ Yes ◯ No

Do you believe that your education was interrupted as a result of your diagnosis?

◯ Yes ◯ No

Did your diagnosis prevent you from completing education?

◯ Yes ◯ No

Does your condition mean that you have a carer?

◯ Yes ◯ No

If yes, is that person a member of family or a professionally paid carer?

◯ Family member
◯ Professional carer
◯ Other

Have you accessed support from a charity for your condition?

◯ Yes ◯ No

If yes, which one?

**Patient Health Questionnaire 4 Over the last two weeks, how often have you been bothered by the following problems?**

Feeling nervous, anxious or on edge?

◯ Not at all
◯ Several Days
◯ More than half the days
◯ Nearly every day

Not being able to stop or control worrying?

◯ Not at all
◯ Several Days
◯ More than half the days
◯ Nearly every day

Feeling down, depressed or hopeless?

◯ Not at all
◯ Several Days
◯ More than half the days
◯ Nearly every day

Little interest or pleasure in doing things?

◯ Not at all
◯ Several Days
◯ More than half the days
◯ Nearly every day

**EQ-5D-5L**

**Under each heading, please tick the ONE box that best describes your health TODAY.**

Mobility

◯ I have no problems in walking about
◯ I have slight problems in walking about
◯ I have moderate problems in walking about
◯ I have severe problems in walking about
◯ I am unable to walk about

Self-Care

◯ I have no problems washing or dressing myself
◯ I have slight problems washing or dressing myself
◯ I have moderate problems washing or dressing myself
◯ I have severe problems washing or dressing myself
◯ I am unable to wash or dress myself

Usual Activities
(e.g. work, study, housework, family or leisure activities)

◯ I have no problems doing my usual activities
◯ I have slight problems doing my usual activities
◯ I have moderate problems doing my usual activities
◯ I have severe problems doing my usual activities
◯ I am unable to do my usual activities

Pain/Discomfort

◯ I have no pain or discomfort
◯ I have slight pain or discomfort
◯ I have moderate pain or discomfort
◯ I have severe pain or discomfort
◯ I have extreme pain or discomfort

Anxiety/Depression

◯ I am not anxious or depressed
◯ I am slightly anxious or depressed
◯ I am moderately anxious or depressed
◯ I am severely anxious or depressed
◯ I am extremely anxious or depressed

Please rate your health score today, with 0 being the worst health you can imagine and 100 being the best health you can imagine.

0 50 100

*(Place a mark on the scale above)*

## Hypermobility and Neurodiversity

Recent work has found that more than 50% of participants with a neurodivergent diagnosis demonstrated elevated levels of hypermobility, compared with just 20% of participants in the general population.

The following questionnaire aims to explore the prevalence of neurodivergence in a population with a hypermobile diagnosis. This questionnaire will add approximately ten minutes to the total survey time and therefore it is completely optional. If you would like to take part in this additional research, please indicate "Yes" below. If not, please indicate "No", and move on to the final section.

◯ Yes ◯ No

---

A score of 26 and above in the folowing questionnaire could be considered an indication of Autism Spectrum Disorder (ASD) but it is important to note that this questionnaire is not a diagnostic tool and that a high score does not always indicate the presence of an ASD.

If you would like to see the results at the end of your questionnaire, please indicate "Yes" below. Otherwise, please indicate "No" and these will remain hidden.

◯ Yes ◯ No

---

I prefer to do things with others, rather than on my own.

◯ Definitely Agree
◯ Slightly Agree
◯ Slightly Disagree
◯ Definitely Disagree

---

I prefer to do things the same way over and over again.

◯ Definitely Agree
◯ Slightly Agree
◯ Slightly Disagree
◯ Definitely Disagree

---

If I try to imagine something, I find it very easy to create a picture in my mind.

◯ Definitely Agree
◯ Slightly Agree
◯ Slightly Disagree
◯ Definitely Disagree

---

I frequently get so strongly absorbed in one thing that I lose sight of other things.

◯ Definitely Agree
◯ Slightly Agree
◯ Slightly Disagree
◯ Definitely Disagree

---

I often notice small sounds when others do not.

◯ Definitely Agree
◯ Slightly Agree
◯ Slightly Disagree
◯ Definitely Disagree

I usually notice car number plates or similar strings of information.

◯ Definitely Agree
◯ Slightly Agree
◯ Slightly Disagree
◯ Definitely Disagree

Other people frequently tell me that what I've said is impolite, even though I think it is polite.

◯ Definitely Agree
◯ Slightly Agree
◯ Slightly Disagree
◯ Definitely Disagree

When I'm reading a story, I can easily imagine what the characters might look like.

◯ Definitely Agree
◯ Slightly Agree
◯ Slightly Disagree
◯ Definitely Disagree

I am fascinated by dates.

◯ Definitely Agree
◯ Slightly Agree
◯ Slightly Disagree
◯ Definitely Disagree

In a social group, I can easily keep track of several different people's conversations.

◯ Definitely Agree
◯ Slightly Agree
◯ Slightly Disagree
◯ Definitely Disagree

I find social situations easy.

◯ Definitely Agree
◯ Slightly Agree
◯ Slightly Disagree
◯ Definitely Disagree

I tend to notice details that others do not.

◯ Definitely Agree
◯ Slightly Agree
◯ Slightly Disagree
◯ Definitely Disagree

I would rather go to a library than a party.

◯ Definitely Agree
◯ Slightly Agree
◯ Slightly Disagree
◯ Definitely Disagree

I find making up stories easy.

◯ Definitely Agree
◯ Slightly Agree
◯ Slightly Disagree
◯ Definitely Disagree

I find myself drawn more strongly to people than to things.

◯ Definitely Agree
◯ Slightly Agree
◯ Slightly Disagree
◯ Definitely Disagree

I tend to have very strong interests which I get upset about if I can't pursue.

◯ Definitely Agree
◯ Slightly Agree
◯ Slightly Disagree
◯ Definitely Disagree

I enjoy social chit-chat.

◯ Definitely Agree
◯ Slightly Agree
◯ Slightly Disagree
◯ Definitely Disagree

When I talk, it isn't always easy for others to get a word in edgeways.

◯ Definitely Agree
◯ Slightly Agree
◯ Slightly Disagree
◯ Definitely Disagree

I am fascinated by numbers.

◯ Definitely Agree
◯ Slightly Agree
◯ Slightly Disagree
◯ Definitely Disagree

When I'm reading a story, I find it difficult to work out the characters' intentions.

◯ Definitely Agree
◯ Slightly Agree
◯ Slightly Disagree
◯ Definitely Disagree

I don't particularly enjoy reading fiction.

◯ Definitely Agree
◯ Slightly Agree
◯ Slightly Disagree
◯ Definitely Disagree

I find it hard to make new friends.

◯ Definitely Agree
◯ Slightly Agree
◯ Slightly Disagree
◯ Definitely Disagree

I notice patterns in things all the time.

◯ Definitely Agree
◯ Slightly Agree
◯ Slightly Disagree
◯ Definitely Disagree

I would rather go to the theatre than a museum.

◯ Definitely Agree
◯ Slightly Agree
◯ Slightly Disagree
◯ Definitely Disagree

It does not upset me if my daily routine is disturbed.

◯ Definitely Agree ◯ Slightly Agree ◯ Slightly Disagree ◯ Definitely Disagree

I frequently find that I don't know how to keep a conversation going.

◯ Definitely Agree
◯ Slightly Agree
◯ Slightly Disagree
◯ Definitely Disagree

I find it easy to "read between the lines" when someone is talking to me.

◯ Definitely Agree
◯ Slightly Agree
◯ Slightly Disagree
◯ Definitely Disagree

I usually concentrate more on the whole picture, rather than the small details.

◯ Definitely Agree
◯ Slightly Agree
◯ Slightly Disagree
◯ Definitely Disagree

I am not very good at remembering phone numbers.

◯ Definitely Agree
◯ Slightly Agree
◯ Slightly Disagree
◯ Definitely Disagree

I often don't notice small changes in a situation, or a person's appearance.

◯ Definitely Agree
◯ Slightly Agree
◯ Slightly Disagree
◯ Definitely Disagree

I know how to tell if someone listening to me is getting bored.

◯ Definitely Agree
◯ Slightly Agree
◯ Slightly Disagree
◯ Definitely Disagree

I find it easy to do more than one thing at once.

◯ Definitely Agree
◯ Slightly Agree
◯ Slightly Disagree
◯ Definitely Disagree

When I talk on the phone, I'm not sure when it's my turn to speak.

◯ Definitely Agree
◯ Slightly Agree
◯ Slightly Disagree
◯ Definitely Disagree

I enjoy doing things spontaneously.

◯ Definitely Agree
◯ Slightly Agree
◯ Slightly Disagree
◯ Definitely Disagree

I am often the last to understand the point of a joke.

◯ Definitely Agree
◯ Slightly Agree
◯ Slightly Disagree
◯ Definitely Disagree

I find it easy to work out what someone is thinking or feeling just by looking at their face.

◯ Definitely Agree
◯ Slightly Agree
◯ Slightly Disagree
◯ Definitely Disagree

If there is an interruption, I can switch back to what I was doing very quickly.

◯ Definitely Agree
◯ Slightly Agree
◯ Slightly Disagree
◯ Definitely Disagree

I am good at social chit-chat.

◯ Definitely Agree
◯ Slightly Agree
◯ Slightly Disagree
◯ Definitely Disagree

People often tell me that I keep going on and on about the same thing.

◯ Definitely Agree
◯ Slightly Agree
◯ Slightly Disagree
◯ Definitely Disagree

When I was young, I used to enjoy playing games involving pretending with other children.

◯ Definitely Agree
◯ Slightly Agree
◯ Slightly Disagree
◯ Definitely Disagree

I like to collect information about categories of things (e.g. types of car, types of bird, types of train, types of plant, etc.).

◯ Definitely Agree
◯ Slightly Agree
◯ Slightly Disagree
◯ Definitely Disagree

I find it difficult to imagine what it would be like to be someone else.

◯ Definitely Agree
◯ Slightly Agree
◯ Slightly Disagree
◯ Definitely Disagree

I like to plan any activities I participate in carefully.

◯ Definitely Agree
◯ Slightly Agree
◯ Slightly Disagree
◯ Definitely Disagree

I enjoy social occasions.

◯ Definitely Agree
◯ Slightly Agree
◯ Slightly Disagree
◯ Definitely Disagree

I find it difficult to work out people's intentions.

◯ Definitely Agree
◯ Slightly Agree
◯ Slightly Disagree
◯ Definitely Disagree

New situations make me anxious.

◯ Definitely Agree
◯ Slightly Agree
◯ Slightly Disagree
◯ Definitely Disagree

I enjoy meeting new people.

◯ Definitely Agree
◯ Slightly Agree
◯ Slightly Disagree
◯ Definitely Disagree

I am a good diplomat.

◯ Definitely Agree
◯ Slightly Agree
◯ Slightly Disagree
◯ Definitely Disagree

I am not very good at remembering people's date of birth.

◯ Definitely Agree
◯ Slightly Agree
◯ Slightly Disagree
◯ Definitely Disagree

I find it very easy to play games with children that involve pretending.

◯ Definitely Agree
◯ Slightly Agree
◯ Slightly Disagree
◯ Definitely Disagree

Your AQ Score:

______________________________
(This score is only valid if you have answered all of the questions in this section.)

**Need for a Specialist Centre in Scotland The boxes below will only appear if you currently live in Scotland:**

Do you believe there is value in a specialist centre for the diagnosis and treatment of HSD/hEDS patients in Scotland?

○ Yes ○ No

Do you believe there is value in a specialist centre for the training and education of Heath Care Professionals in Scotland?

○ Yes ○ No

We would be very happy to hear your thoughts on the value of a specialist service in Scotland. If you have time, please leave a comment.

## Future Research

We would be interested to hear more about the lived experience of your diagnosis. If you have time, please leave some comments in the box below.
Some things you might want to talk about are your experiences of the healthcare system or the impact of your diagnosis on your life. These can be both positive or negative, all feedback is welcome!

Thanks for completing this short survey. If you do not want to consider taking part in future research, please indicate "no" using the button below, go on to the following page and click "submit". This will electronically sign this form and mark the end of the survey. It will also confirm that you consent to the study team storing the data you have provided. Further information on how we store and use personal data you have provided for our research can be found here.

1. I agree to be contacted about ethically approved research studies relevant to hypermobile Ehlers-Danlos Syndrome and Hypermobile Spectrum Disorder. I understand that agreeing to be contacted does not oblige me to participate in any further studies.

◯ Yes ◯ No

2. I understand that registering my interest into future research will involve me providing personal details for administrative reasons so that the study team can get in touch.

◯ Yes ◯ No

If you answered "Yes" to the questions above, please provide your contact details so that a member of the study team can get in touch:

First name: ______________________

Surname: ______________________

Email address: ______________________

Telephone number: ______________________
