# Supplementary material for: Health experiences and outcomes of autistic and non-autistic adults with hypermobile Ehlers-Danlos syndrome and hypermobility spectrum disorder
Source: BMC Med. 2026 Feb 25;24:193. doi: 10.1186/s12916-026-04713-2 (PMC13040810; doi:10.1186/s12916-026-04713-2)
Supplement: Supplementary file 2 — Additional file 2: Additional statistical detail, model comparison, and figures supplemental to the main text. [file 12916_2026_4713_MOESM2_ESM.docx]

**Supplement 2: hEDS / HSD associated symptoms**

This supplement relates the data presented in Figure 1.

Supplement 2, Table 1: hEDS/HSD associated symptoms, % (n) within groups; overall & pairwise χ² tests, with Bonferroni-corrections

|  | **Non-Autistic** | **High AQ** | | **Autistic** | **χ²** | | **df** | | | **p (overall)** | **Cramer’s V** | | **Comparison** | |
| --- | --- | --- | --- | --- | --- | --- | --- | --- | --- | --- | --- | --- | --- | --- |
|  | | | | | | | | | | | | | | |
| **Dermatological** | | | | | | | | | | | | | | |
| Abnormal scarring | 38.1% (338) | 38.3% (165) | | 48.6% (212) | 14.85 | | 2 | | | 0.001 | 0.092 | | Aut > Non Aut***  Aut > High AQ** | |
| Poor wound healing | 41.6% (369) | 43.4% (187) | | 49.3% (215) | 7.13 | | 2 | | | 0.028 | 0.064 | | Aut > Non Aut* | |
| Stretchy skin | 39.5% (350) | 44.5% (192) | | 47.9% (209) | 9.28 | | 2 | | | 0.010 | 0.073 | | Aut > Non Aut* | |
| **Orthopaedic** | | | | | | | | | | | | | | |
| Joint Dislocations | 41.3% (366) | 47.8% (206) | | 54.4% (237) | 20.82 | | 2 | | | 0.000 | 0.109 | | Aut > Non Aut*** | |
| Joint Subluxations | 72.2% (640) | 73.8% (318) | | 79.6% (347) | 8.60 | | 2 | | | 0.014 | 0.070 | | Aut > Non Aut* | |
| **Internal medicine** | | | | | | | | | | | | | | |
| Abdominal hernia(s) | 10.1% (90) | 11.1% (48) | | 10.6% (46) | 0.31 | | 2 | | | 0.858 | 0.013 | | ns | |
| Pelvic prolapse | 14.4% (128) | 12.8% (55) | | 12.2% (53) | 1.54 | | 2 | | | 0.464 | 0.030 | | ns | |
| **Cardiological** | | | | | | | | | | | | | | |
| Mitral valve prolapse | 7.4% (66) | 6.3% (27) | | 5.5% (24) | 1.91 | | 2 | | | 0.385 | 0.033 | | ns | |
| **Other** | | | | | | | | | | | | | | |
| Chronic pain | 81.3% (721) | 85.3% (369) | 89% (388) | | | 13.88 | | 2 | 0.001 | | | 0.089 | | Aut > Non Aut** |

* p<.05; ** p<.01; *** p<.001; “ns” = non-significant

Supplement 2, Table 2: Pairwise X^2^ comparisons of hEDS/HSD associated symptoms across groups

|  | **Autistic vs High AQ** | **Autistic vs Non-autistic** | **High AQ vs Non-autistic** |
| --- | --- | --- | --- |
| **Dermatological** | | | |
| Abnormal scarring | 48.7% vs 38.9%, p = 0.006, V = 0.10 | 48.7% vs 38.9%, p = < .001, V = 0.10 | 38.9% vs 38.9%, p = 1.000, V = 0.00 |
| Poor wound healing | 49.1% vs 43.8%, p = 0.241, V = 0.06 | 49.1% vs 42.5%, p = 0.024, V = 0.07 | 43.8% vs 42.5%, p = 1.000, V = 0.02 |
| Stretchy skin | 48.1% vs 45.2%, p = 0.951, V = 0.03 | 48.1% vs 39.5%, p = 0.010, V = 0.08 | 45.2% vs 39.5%, p = 0.235, V = 0.05 |
| **Orthopaedic** | | | |
| Joint Dislocations | 54.3% vs 47.6%, p = 0.160, V = 0.07 | 54.3% vs 42.2%, p = < .001, V = 0.12 | 47.6% vs 42.2%, p = 0.074, V = 0.06 |
| Joint Subluxations | 79.1% vs 73.3%, p = 0.130, V = 0.07 | 79.1% vs 71.2%, p = 0.011, V = 0.08 | 73.3% vs 71.2%, p = 1.000, V = 0.02 |
| **Internal medicine** | | | |
| Abdominal hernia(s) | 11.0% vs 11.7%, p = 1.000, V = 0.01 | 11.0% vs 11.1%, p = 1.000, V = 0.01 | 11.7% vs 11.1%, p = 1.000, V = 0.02 |
| Pelvic prolapse | 12.7% vs 13.3%, p = 1.000, V = 0.01 | 12.7% vs 15.5%, p = 0.774, V = 0.03 | 13.3% vs 15.5%, p = 1.000, V = 0.02 |
| **Cardiological** | | | |
| Mitral valve prolapse | 5.8% vs 6.7%, p = 1.000, V = 0.02 | 5.8% vs 8.3%, p = 0.567, V = 0.04 | 6.7% vs 8.3%, p = 1.000, V = 0.02 |
| **Other** | | | |
| Chronic pain | 88.6% vs 85.3%, p = 0.405, V = 0.05 | 88.6% vs 80.1%, p = 0.001, V = 0.10 | 85.3% vs 80.1%, p = 0.154, V = 0.05 |

* p<.05; ** p<.01; *** p<.001; “ns” = non-significant

| **Supplement 3: Co-occurring conditions**  This supplement relates the data presented in Figure 2.  Supplement 3: Table 1A: Co-occurring physical health conditions: % (n) within groups; overall & pairwise χ² tests, with Bonferroni corrections   \|  \| **Non-autistic** \| **High AQ** \| **Autistic** \| **χ²** \| **df** \| **p (overall)** \| **Cramer’s V** \| **Comparison** \| \| --- \| --- \| --- \| --- \| --- \| --- \| --- \| --- \| --- \| \| Adrenal dysfunction \| 4.6% (41) \| 2.8% (12) \| 5.5% (24) \| 4.05 \| 2 \| 0.132 \| 0.048 \| ns \| \| Alpha-1 AT deficiency \| 0.3% (3) \| 0.7% (3) \| 0.5% (2) \| 0.82 \| 2 \| 0.664 \| 0.022 \| ns \| \| Bleeding/clotting \| 13.6% (121) \| 13.0% (56) \| 14.2% (62) \| 0.28 \| 2 \| 0.87 \| 0.013 \| ns \| \| Dysautonomia/POTS \| 42.5% (377) \| 43.6% (188) \| 57.3% (250) \| 27.73 \| 2 \| 0 \| 0.126 \| Aut > Non Aut***  Aut > High AQ*** \| \| Endometriosis \| 17.7% (157) \| 16.2% (70) \| 17.7% (77) \| 0.47 \| 2 \| 0.789 \| 0.016 \| ns \| \| Gastrointestinal manifestations (e.g. gastroparesis, IBS, GORD) \| 65.8% (584) \| 66.6% (287) \| 71.3% (311) \| 4.18 \| 2 \| 0.124 \| 0.049 \| ns \| \| Mast Cell Activation Syndrome \| 17.8% (158) \| 16.7% (72) \| 25.0% (109) \| 12.2 \| 2 \| 0.002 \| 0.083 \| Aut > Non Aut**;  Aut > High AQ* \| \| Porphyria \| 0.3% (3) \| 0.2% (1) \| 0.7% (3) \| 1.3 \| 2 \| 0.522 \| 0.027 \| ns \| \| Sleep disturbances \| 18.2% (161) \| 22.5% (97) \| 29.6% (129) \| 22.3 \| 2 \| 0 \| 0.113 \| Aut > Non Aut*** \| \| Raynaud’s \| 27.3% (242) \| 30.4% (131) \| 39.2% (171) \| 19.57 \| 2 \| 0 \| 0.106 \| Aut > Non Aut***  Aut > High AQ* \|   * p<.05; ** p<.01; *** p<.001; “ns” = non-significant  Supplement 3: Table 1B: Co-occurring neurodivergence, neurological and mental health conditions, % (n) within groups; overall & pairwise χ² tests with Bonferroni corrections   \|  \| **Non-autistic** \| **High AQ** \| **Autistic** \| **χ²** \| **df** \| **p (overall)** \| **Cramer’s V** \| **Comparison** \| \| --- \| --- \| --- \| --- \| --- \| --- \| --- \| --- \| --- \| \| **Neurological** \| \| \| \| \| \| \| \| \| \| CCI/AAI \| 6.8% (60) \| 5.6% (24) \| 8.5% (37) \| 2.92 \| 2 \| 0.232 \| 0.041 \| ns \| \| Chiari malformation \| 1.5% (13) \| 2.6% (11) \| 2.8% (12) \| 3.12 \| 2 \| 0.21 \| 0.042 \| ns \| \| Migraine \| 52.6% (467) \| 58.5% (252) \| 54.4% (237) \| 3.97 \| 2 \| 0.138 \| 0.048 \| ns \| \| Tethered cord syndrome \| 1.0% (9) \| 0.2% (1) \| 0.7% (3) \| 2.44 \| 2 \| 0.296 \| 0.037 \| ns \| \| ME/CFS \| 30.4% (270) \| 27.6% (119) \| 39.0% (170) \| 14.62 \| 2 \| 0.001 \| 0.091 \| Aut > Non Aut**  Aut > High AQ** \| \| **Mental health** \| \| \| \| \| \| \| \| \| \| Anxiety \| 66.0% (585) \| 74.9% (323) \| 86.7% (378) \| 65.07 \| 2 \| 0 \| 0.193 \| Aut > Non Aut***  Aut > High AQ***  High AQ > Non Aut** \| \| Depression \| 59.1% (524) \| 68.7% (296) \| 75.7% (330) \| 38.18 \| 2 \| 0 \| 0.147 \| Aut > Non Aut***  High AQ > Non Aut** \| \| **Neurodivergence** \|  \|  \|  \|  \|  \|  \|  \|  \| \| ADHD \| 11.3% (100) \| 13.7% (59) \| 44.0% (192) \| 210.29 \| 2 \| 0 \| 0.346 \| Aut > Non Aut***  Aut > High AQ*** \| \| Dyscalculia° \| 3.9% (35) \| 2.8% (12) \| 10.8% (47) \| 36.49 \| 2 \| 0 \| 0.184 \| Aut > Non Aut***  Aut > High AQ*** \| \| Dyslexia° \| 8.8% (78) \| 9.5% (41) \| 15.6% (68) \| 16.84 \| 2 \| 0 \| 0.125 \| Aut > Non Aut***  Aut > High AQ* \| \| Dyspraxia/DCD° \| 3.2% (28) \| 5.8% (25) \| 11.2% (49) \| 36.88 \| 2 \| 0 \| 0.185 \| Aut > Non Aut***  Aut > High AQ* \| \| *1Pairwise p-values (Autistic vs No Autism; Autistic vs High AQ; High AQ vs No Autism) were Bonferroni-adjusted across the three contrasts.* \| \| \| \| \| \| \| \| \|   ° These data should be interpreted with caution, as 39% of participants were not presented with these conditions on the form due to an error in the online survey. However, the proportion of missing data was distributed equally across the three groups (χ²(2) = 0.13, p = .94)  * p<.05; ** p<.01; *** p<.001; “ns” = non-significant  Supplement 3: Table 2A: Pairwise X^2^ comparisons of co-occurring physical health conditions across groups.   \| **Condition** \| **Autistic Vs High AQ** \| **Autistic Vs Non-Autistic** \| **High AQ Vs Non-Autistic** \| \| --- \| --- \| --- \| --- \| \| Adrenal dysfunction \| 5.8% vs 3.0%, p = 0.134, V = 0.07 \| 5.8% vs 5.2%, p = 1.000, V = 0.02 \| 3.0% vs 5.2%, p = 0.333, V = 0.04 \| \| Alpha-1 antitrypsin deficiency \| 0.5% vs 0.7%, p = 1.000, V = 0.02 \| 0.5% vs 0.4%, p = 1.000, V = 0.01 \| 0.7% vs 0.4%, p = 1.000, V = 0.02 \| \| Bleeding or clotting problems \| 14.5% vs 13.3%, p = 1.000, V = 0.02 \| 14.5% vs 14.6%, p = 1.000, V = 0.01 \| 13.3% vs 14.6%, p = 1.000, V = 0.01 \| \| Dysautonomia/ POTS \| 57.2% vs 44.0%, p = < .001, V = 0.14 \| 57.2% vs 43.1%, p = < .001, V = 0.14 \| 44.0% vs 43.1%, p = 1.000, V = 0.01 \| \| Endometriosis \| 18.4% vs 16.8%, p = 1.000, V = 0.02 \| 18.4% vs 19.2%, p = 1.000, V = 0.00 \| 16.8% vs 19.2%, p = 1.000, V = 0.02 \| \| Gastrointestinal manifestations (e.g. gastroparesis, IBS, GORD) \| 70.5% vs 66.3%, p = 0.393, V = 0.05 \| 70.5% vs 65.1%, p = 0.134, V = 0.06 \| 66.3% vs 65.1%, p = 1.000, V = 0.01 \| \| Mast Cell Activation Syndrome (MCAS) \| 25.4% vs 17.4%, p = 0.008, V = 0.10 \| 25.4% vs 19.2%, p = 0.007, V = 0.08 \| 17.4% vs 19.2%, p = 1.000, V = 0.01 \| \| Porphyria \| 0.7% vs 0.2%, p = 0.966, V = 0.03 \| 0.7% vs 0.4%, p = 1.000, V = 0.02 \| 0.2% vs 0.4%, p = 1.000, V = 0.01 \| \| Raynaud's phenomenon \| 39.3% vs 31.5%, p = 0.019, V = 0.09 \| 39.3% vs 28.8%, p = < .001, V = 0.12 \| 31.5% vs 28.8%, p = 0.717, V = 0.03 \| \| Sleep disturbances (e.g. sleep apnea, narcolepsy) \| 30.0% vs 23.5%, p = 0.053, V = 0.08 \| 30.0% vs 19.2%, p = < .001, V = 0.13 \| 23.5% vs 19.2%, p = 0.185, V = 0.05 \|   * p<.05; ** p<.01; *** p<.001; “ns” = non-significant | | | |
| --- | --- | --- | --- | --- | --- | --- | --- | --- | --- | --- | --- | --- | --- | --- | --- | --- | --- | --- | --- | --- | --- | --- | --- | --- | --- | --- | --- | --- | --- | --- | --- | --- | --- | --- | --- | --- | --- | --- | --- | --- | --- | --- | --- | --- | --- | --- | --- | --- | --- | --- | --- | --- | --- | --- | --- | --- | --- | --- | --- | --- | --- | --- | --- | --- | --- | --- | --- | --- | --- | --- | --- | --- | --- | --- | --- | --- | --- | --- | --- | --- | --- | --- | --- | --- | --- | --- | --- | --- | --- | --- | --- | --- | --- | --- | --- | --- | --- | --- | --- | --- | --- | --- | --- | --- | --- | --- | --- | --- | --- | --- | --- | --- | --- | --- | --- | --- | --- | --- | --- | --- | --- | --- | --- | --- | --- | --- | --- | --- | --- | --- | --- | --- | --- | --- | --- | --- | --- | --- | --- | --- | --- | --- | --- | --- | --- | --- | --- | --- | --- | --- | --- | --- | --- | --- | --- | --- | --- | --- | --- | --- | --- | --- | --- | --- | --- | --- | --- | --- | --- | --- | --- | --- | --- | --- | --- | --- | --- | --- | --- | --- | --- | --- | --- | --- | --- | --- | --- | --- | --- | --- | --- | --- | --- | --- | --- | --- | --- | --- | --- | --- | --- | --- | --- | --- | --- | --- | --- | --- | --- | --- | --- | --- | --- | --- | --- | --- | --- | --- | --- | --- | --- | --- | --- | --- | --- | --- | --- | --- | --- | --- | --- | --- | --- | --- | --- | --- | --- | --- | --- | --- | --- | --- | --- | --- | --- | --- | --- | --- | --- | --- | --- | --- | --- | --- | --- | --- | --- | --- | --- | --- | --- | --- | --- | --- | --- | --- | --- | --- | --- | --- | --- | --- | --- | --- | --- | --- | --- | --- | --- | --- | --- | --- | --- | --- | --- | --- | --- | --- | --- | --- |
| Supplement 3: Table 2B: Pairwise X^2^ comparisons of co-occurring neurodivergence, neurological and mental health conditions across groups.   \|  \| **Autistic vs High AQ** \| **Autistic Vs Non-autistic** \| **High AQ Vs Non-Autistic** \| \| --- \| --- \| --- \| --- \| \| **Neurological** \| \| \| \| \| Chiari malformation \| 2.9% vs 2.7%, p = 1.000, V = 0.01 \| 2.9% vs 1.6%, p = 0.318, V = 0.04 \| 2.7% vs 1.6%, p = 0.498, V = 0.04 \| \| CCI/AAI \| 8.9% vs 5.9%, p = 0.279, V = 0.06 \| 8.9% vs 7.5%, p = 0.777, V = 0.03 \| 5.9% vs 7.5%, p = 1.000, V = 0.02 \| \| ME/CFS \| 38.9% vs 27.7%, p = 0.001, V = 0.12 \| 38.9% vs 31.1%, p = 0.006, V = 0.09 \| 27.7% vs 31.1%, p = 0.873, V = 0.03 \| \| Migraine \| 54.5% vs 57.9%, p = 0.666, V = 0.04 \| 54.5% vs 53.0%, p = 1.000, V = 0.02 \| 57.9% vs 53.0%, p = 0.140, V = 0.05 \| \| **Mental Health** \| \| \| \| \| Anxiety \| 86.2% vs 74.4%, p = < .001, V = 0.15 \| 86.2% vs 65.3%, p = < .001, V = 0.22 \| 74.4% vs 65.3%, p = 0.003, V = 0.09 \| \| Depression \| 75.2% vs 68.1%, p = 0.064, V = 0.08 \| 75.2% vs 58.7%, p = < .001, V = 0.16 \| 68.1% vs 58.7%, p = 0.002, V = 0.09 \| \| **Neurodivergence** \| \| \| \| \| ADHD \| 43.5% vs 14.4%, p = < .001, V = 0.34 \| 43.5% vs 12.5%, p = < .001, V = 0.37 \| 14.4% vs 12.5%, p = 0.621, V = 0.03 \| \| Dyscalculia \| 10.80% vs 2.80%, p = < .001, V = 0.21 \| 10.80% vs 3.90%, p = < .001, V = 0.18 \| 2.80% vs 3.90%, p = 0.792, V = 0.04 \| \| Dyslexia \| 15.60% vs 9.50%, p = .010, V = 0.13 \| 15.60% vs 8.80%, p = < .001, V = 0.14 \| 9.50% vs 8.80%, p = 1.000, V = 0.01 \| \| Dyspraxia/DCD \| 11.20% vs 5.80%, p = .007, V = 0.13 \| 11.20% vs 3.20%, p = < .001, V = 0.21 \| 5.80% vs 3.20%, p = 0.068, V = 0.08 \|   * p<.05; ** p<.01; *** p<.001; “ns” = non-significant | | | |
|  |  |  |  |
|  |  |  |  |
|  |  |  |  |

**Supplement 4:** Services referred to within the past 5 years, with pairwise χ² tests with Bonferroni corrections

Supplement 4: Table 1: Pairwise X^2^ comparisons of referral to clinical services by group.

| Service | **Autistic vs High-AQ** | **Autistic vs No Autism** | **High-AQ vs No Autism** |
| --- | --- | --- | --- |
| Cardiology | 43.7% vs 36.0%, *p* < .001, *V* = .08 | 43.7% vs 33.3%, *p* < .001, *V* = .10 | 36.0% vs 33.3%, *p* = .26, *V* = .03 |
| Clinical Psychology | 18.7% vs 12.6%, *p* < .001, *V* = .08 | 18.7% vs 9.9%, *p* < .001, *V* = .12 | 12.6% vs 9.9%, *p* = .03, *V* = .04 |
| Gastroenterology | 36.9% vs 28.6%, *p* < .001, *V* = .09 | 36.9% vs 26.9%, *p* < .001, *V* = .10 | 28.6% vs 26.9%, *p* = .78, *V* = .02 |
| Gynaecology | 22.7% vs 24.3%, *p* = 1.00, *V* = .02 | 22.7% vs 21.7%, *p* = 1.00, *V* = .01 | 24.3% vs 21.7%, *p* = .21, *V* = .03 |
| Neurology | 25.0% vs 23.4%, *p* = 1.00, *V* = .02 | 25.0% vs 19.3%, *p* < .001, *V* = .07 | 23.4% vs 19.3%, *p* = .01, *V* = .05 |
| Occupational Therapy | 28.1% vs 20.3%, *p* < .001, *V* = .09 | 28.1% vs 17.1%, *p* < .001, *V* = .13 | 20.3% vs 17.1%, *p* = .04, *V* = .04 |
| Other | 18.0% vs 18.3%, *p* = 1.00, *V* = .01 | 18.0% vs 19.5%, *p* = .70, *V* = .02 | 18.3% vs 19.5%, *p* = 1.00, *V* = .01 |
| Pain Management | 32.7% vs 29.2%, *p* = .18, *V* = .04 | 32.7% vs 25.0%, *p* < .001, *V* = .08 | 29.2% vs 25.0%, *p* = .01, *V* = .05 |
| Physiotherapy | 64.5% vs 62.6%, *p* = .90, *V* = .02 | 64.5% vs 58.5%, *p* < .001, *V* = .06 | 62.6% vs 58.5%, *p* = .04, *V* = .04 |
| Podiatry | 20.3% vs 16.4%, *p* = .03, *V* = .05 | 20.3% vs 18.1%, *p* = .26, *V* = .03 | 16.4% vs 18.1%, *p* = .58, *V* = .02 |
| Rheumatology | 52.8% vs 46.4%, *p* < .001, *V* = .06 | 52.8% vs 45.0%, *p* < .001, *V* = .07 | 46.4% vs 45.0%, *p* = 1.00, *V* = .01 |
| Urology | 17.6% vs 13.8%, *p* = .02, *V* = .05 | 17.6% vs 13.9%, *p* = .01, *V* = .05 | 13.8% vs 13.9%, *p* = 1.00, *V* = .00 |

**Supplement 6: Figure 1:** *Model Diagnostics for the final health score model*

*Plots assessing the model's statistical assumptions. Top-left (Residuals vs. Fitted): Checks for linearity and homoscedasticity (constant variance). The red line should be flat and the points randomly scattered. Bottom-left (Normal Q-Q): Checks for normality of residuals; points should fall along the dashed line. Top-right (Residuals vs. Leverage): Identifies influential data points. Points with high leverage and large residuals (especially those outside the red dashed Cook's distance lines) can disproportionately affect the model. Bottom-right (Scale-Location): A further check for homoscedasticity; the red line should be flat.*


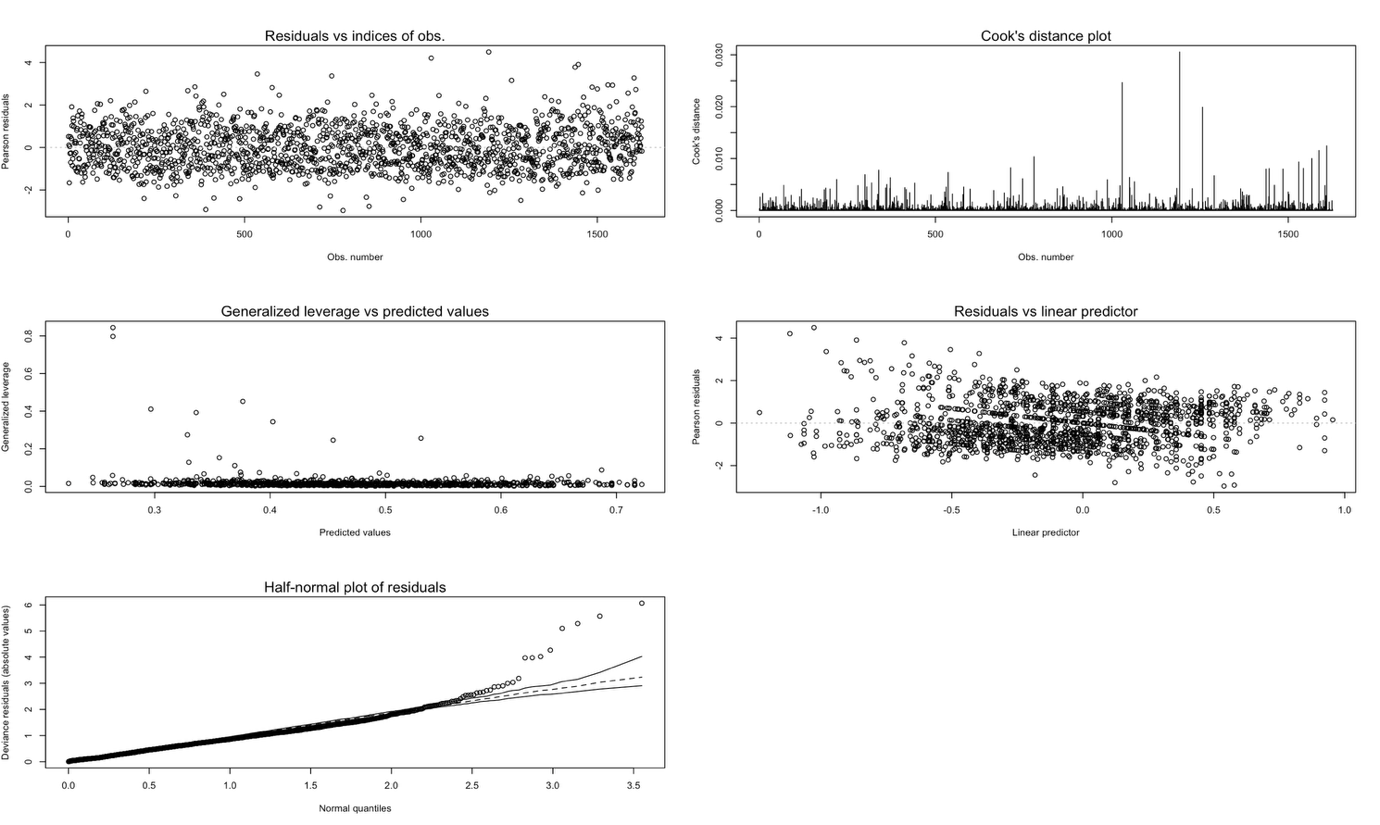


Supplement 7: Figure 1: Model diagnostics for the final self-reported mental health (PHQ) model.

*This figure, generated by the performance package, shows a range of diagnostic plots. Posterior Predictive Check (top-left): The density plot of the observed data (blue) is closely matched by the model's simulated data (green lines), indicating the model generates data that looks like the real data. Linearity (top-right; middle-left): The 'Residuals vs. Fitted' plot and 'Binned Residuals' plot both show that the red smoother line is relatively flat, and the points are randomly scattered, suggesting the linearity assumption is met. Normality of Residuals (middle-right): The Q-Q plot shows points falling closely along the dashed line, confirming the model's residuals are approximately normally distributed. Influential Outliers (bottom-left): No points are flagged as having a high influence (e.g., high Cook's distance) on the model's parameters. Homogeneity of Variance (bottom-right): The 'Scale-Location' plot shows a flat red line, indicating that the variance of the residuals is constant (homoscedastic). Collectively, these diagnostics suggest the model is a good fit for the data and meets its statistical assumptions.*


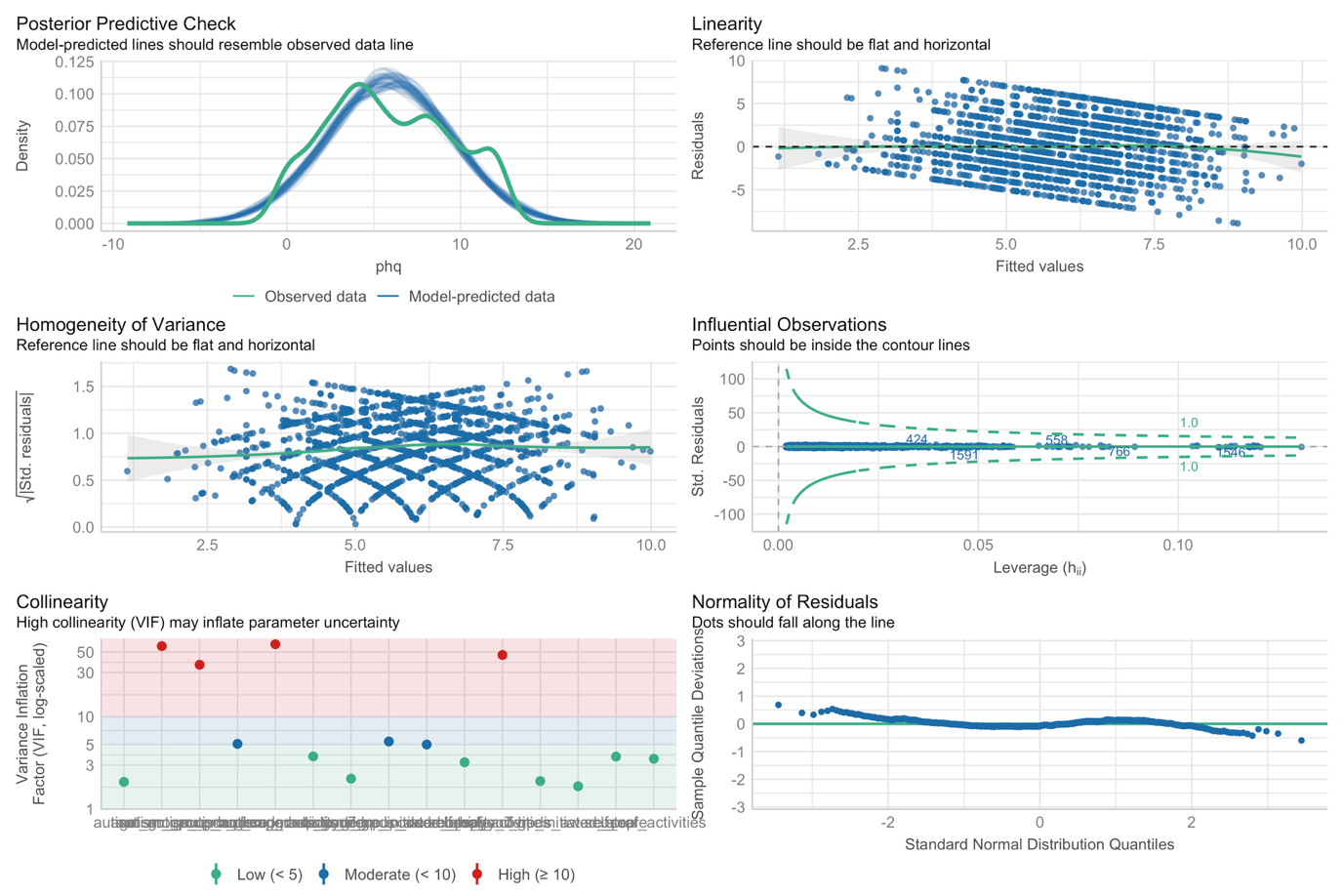


**Supplement 8:** The following section guides the consruction of the Health Score model, including variable selection, model comparisons, and corrections for the selection process (i.e. the LASSO regression).

Supplement 8A: Variable Selection

Supplement 8, Table 1: LASSO-Selected Predictors for the Health Score Model at ₘᵢₙ and λse Penalty Levels

| **Criterion** | | **Selected Predictors** |
| --- | --- | --- |
| **λ**ₘᵢₙ | Joint Dislocations, Gastrointestinal manifestations  (e.g. gastroparesis, IBS, GORD), Attention Deficit Disorder (ADD), Migraine, Cranio-cervical instability  (CCI)/Atlanto-axial instability (AAI), Myalgic  encephalomyelitis/chronic fatigue syndrome (ME/CFS), Bleeding or clotting problems, Chronic pain, has_a_medical_professional, physiptherapist, pain_management, residential_programme, heds_diagnosed, heds_aware_prof, work, have_you_accessed_support, mobility, self_care, usual_activities, pain_discomfort, anxiety_depression, age | |
| **λ_1se_** | mobility, self_care, usual_activities, pain_discomfort, anxiety_depression | |

Note. **λmin** ("Best Fit") is the penalty parameter that minimises cross-validated error, resulting in a model with 22 predictors. **λ1se**

("Most Parsimonious") is the simplest model within one standard error of the minimum, resulting in a model with 5 predictors.

Supplement 8B:

Supplement 8, Table 2: Health Score

We report post-selection inference using the selectiveInference package (Health Score outcome) to adjust for selection-induced bias. However, the package is limited to models including only main effects and does not support interaction terms. As our primary research questions concern group differences and potential interactions between group membership and selected predictors, we instead fit a linear model including interaction terms using the stable λ₁ₛₑ-selected variables.

| Predictor | B | SE | *z* | *p* | 95% CI |
| --- | --- | --- | --- | --- | --- |
| Mobility | -0.03 | 0.01 | -3.88 | < .001 | [-0.04, -0.01] |
| Self care | -0.02 | 0.01 | -2.93 | .003 | [-0.03, -0.01] |
| Usual activities | -0.04 | 0.01 | -6.11 | < .001 | [-0.05, -0.03] |
| Pain discomfort | -0.02 | 0.01 | -2.69 | .007 | [-0.03, -0.01] |
| Anxiety & depression | -0.03 | 0.01 | -6.20 | < .001 | [-0.04, -0.02] |

Supplement 8C:

Supplement 8, Figure 1: Standardized LASSO regression coefficients at the cross‐validated λ_min penalty, predicting self-reported health‐score.

*Bars to the left of zero indicate predictors whose higher values are associated with lower health scores, whereas bars to the right of zero indicate predictors whose presence or higher values are associated with higher health scores.*


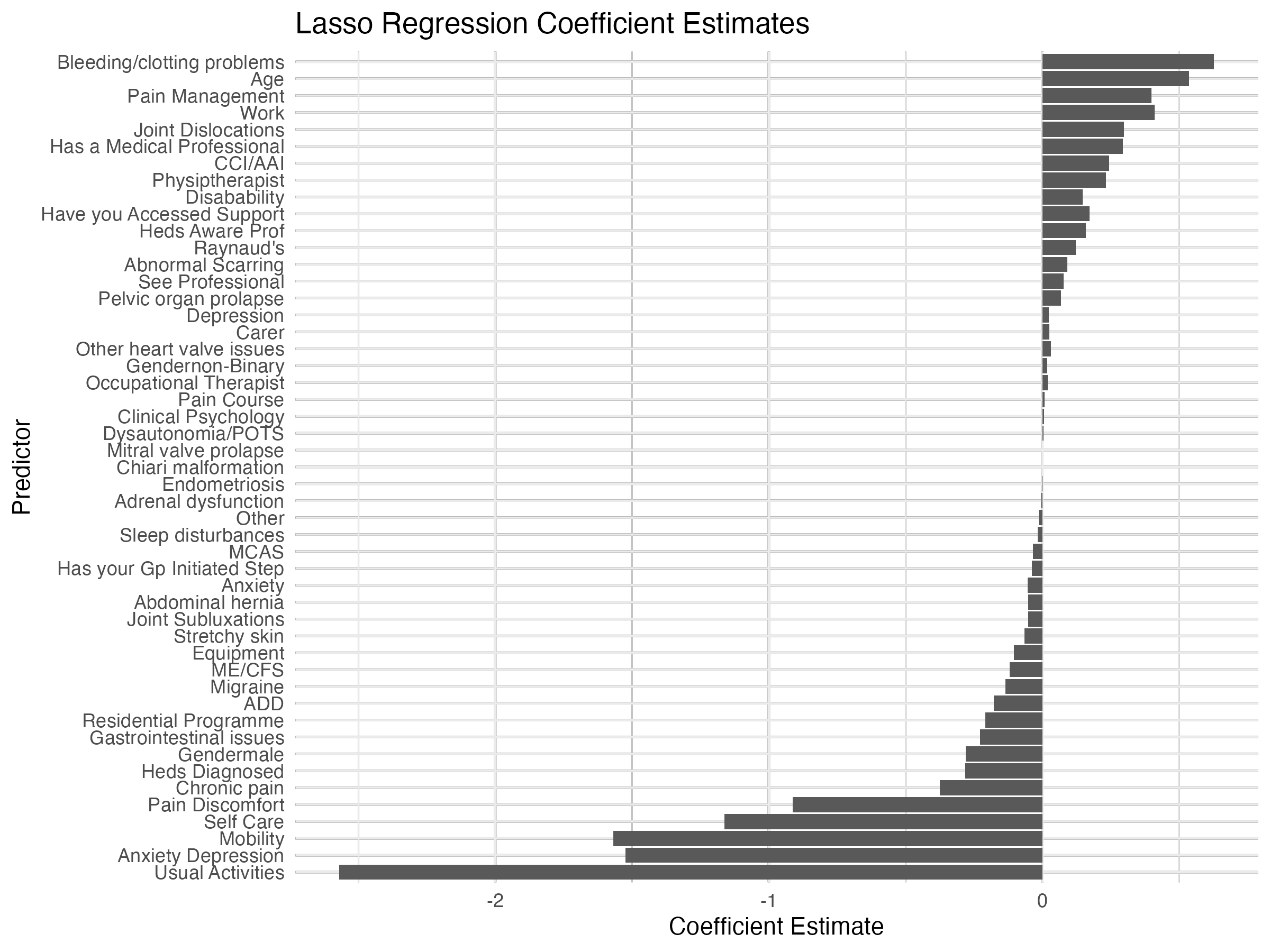


Supplement 8D: Model Comparisons for Health Score

Supplement 8, Table 3: Model Comparisons for Health Score

A series of candidate models were compared for the 0–100 health-score outcome. The beta-regression (betareg) gave the lowest AIC (-999.5), and the highest Akaike weight (ω = .40 each; cumulative ω = .80), comfortably outperforming the ordinary linear model (ΔAIC = 55), the robust linear model (ΔAIC = 71), the ordinal-beta model (ΔAIC = 4), and the zero/one-inflated beta (ΔAIC ≈ 2). Consequently, the beta regression is reported in the manuscript.

| Name | Model | AIC | AIC Weight | AICc | AICc Weight | BIC | BIC Weight | RMSE | Sigma | *R*^2^ |
| --- | --- | --- | --- | --- | --- | --- | --- | --- | --- | --- |
| model_rlm | rlm | -928.72 | 0.00 | -928.24 | 0.00 | -826.23 | 0.00 | 0.18 | 0.18 |  |
| model_lm | lm | -944.87 | 0.00 | -944.40 | 0.00 | -842.39 | 0.00 | 0.18 | 0.18 | .23 |
| model_ordbeta | glmmTMB | -995.46 | 0.05 | -994.89 | 0.05 | -882.19 | 0.00 | 0.18 | 6.34 | .18 |
| model_beta | betareg | -999.46 | 0.40 | -998.99 | 0.40 | -896.98 | 0.49 | 0.18 | 0.94 | .21 |
| model_betaTMB | glmmTMB | -999.46 | 0.40 | -998.99 | 0.40 | -896.98 | 0.49 | 0.18 | 6.34 | .21 |
| model_zibeta | glmmTMB | -997.46 | 0.15 | -996.94 | 0.14 | -889.59 | 0.01 | 0.18 | 6.34 | .72 |

*Note*. AIC: Akaike Information Criterion; AICc: AIC corrected for small sample sizes; AIC Weight: The relative likelihood that the model is the best-fitting model in the set (sums to 1); BIC: Bayesian Information Criterion, a similar metric that more strongly penalises model complexity; BIC Weight: The relative likelihood of the model based on BIC; RMSE: Root Mean Square Error, a measure of the model's average prediction error in the outcome's original units; Residual SD: Residual Standard Deviation (or Sigma); R-squared R2$: A measure of the variance explained by the model. For AIC, AICc, BIC, and RMSE, lower values indicate a better fit. For AIC/BIC Weights, higher values indicate a better fit. The R2 values for non-lm models (e.g., glmmTMB, betareg) are pseudo-R2 and are not directly comparable to the standard R2 from the lm model.

**Supplement 9:** The following section guides the consruction of the PHQ model, including variable selection, model comparisons, and corrections for the selection process (i.e. the LASSO regression).

Supplement 9A: Variable Selection

Table 2: LASSO-Selected Predictors for the PHQ Model at λₘᵢₙ and λse Penalty Levels

| **Criterion** | **Selected Predictors** |
| --- | --- |
| **λ**ₘᵢₙ | Joint Dislocations, Joint Subluxations, Dysautonomia/POTS, ADD (Attention Deficit Disorder), Chiari malformation, Sleep disturbances, ME/CFS (Myalgic Encephalomyelitis/Chronic Fatigue Syndrome), Adrenal dysfunction, Chronic pain, Endometriosis, Other (unspecified comorbidity), GP has initiated steps for care, Diagnosed by a medical professional, Physiotherapist, Occupational Therapist, Currently seeing a professional, Awareness of hEDS by professional, Has accessed support, Clinical Psychology, Has a disability, Self-Care, Usual Activities, Pain/Discomfort, Age, Gender |
| **λ_1se_** | ADD (Attention Deficit Disorder), GP has initiated steps for care, Self-Care, Usual Activities, Pain/Discomfort, Age, Gender |

Note. **λ_min_** ("Best Fit") is the penalty parameter that minimises cross-validated error, resulting in a model with 24 predictors. **λ_1se_**

("Most Parsimonious") is the simplest model within one standard error of the minimum, resulting in a model with 6 predictors.

Supplement 9C: Table 5: We report post-selection inference (PHQ as outcome) using the selectiveInference package to adjust for selection-induced bias. However, the package is limited to models including only main effects and does not support interaction terms. As our primary research questions concern group differences and potential interactions between group membership and selected predictors, we instead fit a linear model including interaction terms using the stable λ₁ₛₑ-selected variables.

| Predictor | *B* | *SE* | 95% *CI* | *z* | *p* |
| --- | --- | --- | --- | --- | --- |
| ADHD | 0.18 | 0.08 | [0.04, 0.31] | 2.32 | .02 |
| GP Initiated Step-Down Care | -0.27 | 0.08 | [-0.40, -0.14] | -3.54 | < .001 |
| Self-Care | 0.24 | 0.1 | [0.05, 0.41] | 2.33 | .02 |
| Usual Activities | 0.31 | 0.11 | [0.14, 0.49] | 2.99 | .003 |
| Pain/Discomfort | 0.81 | 0.09 | [0.66, 0.96] | 8.75 | < .001 |
| Age | -0.6 | 0.08 | [-0.73, -0.47] | -7.66 | < .001 |
| Gender (Non-binary) | 0.22 | 0.08 | [0.08, 0.35] | 2.71 | .007 |

*Supplement 9d: Figure 4* Standardized LASSO regression coefficients at the cross‐validated λ_min penalty, predicting self-reported mental health score. Bars to the left of zero indicate predictors whose higher values are associated with lower health scores, whereas bars to the right of zero indicate predictors whose presence or higher values are associated with higher health scores.


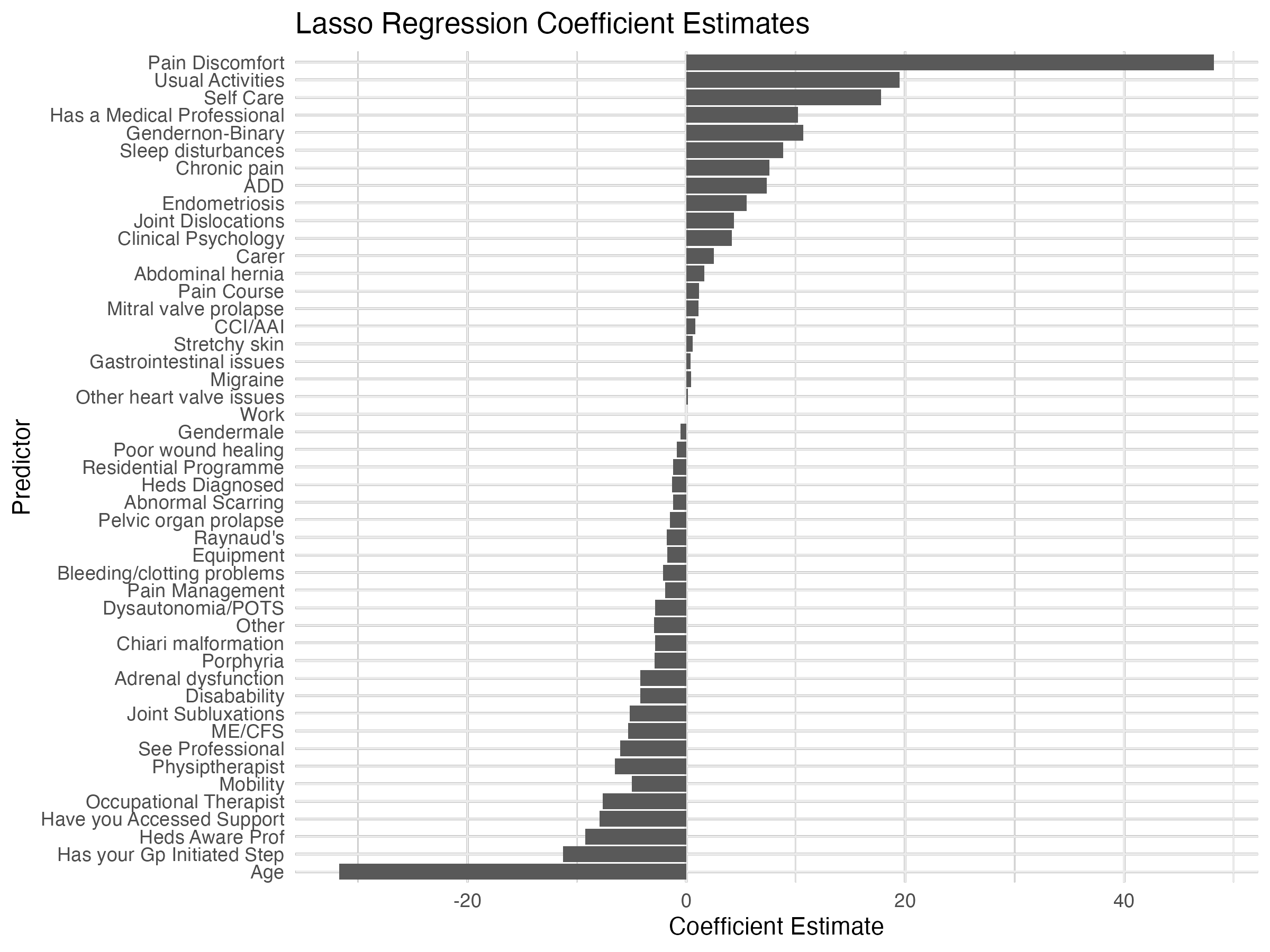


*Supplement 9D: Table 6: Model comparisons for self-reported mental health score.*

Comparison of Model Fit and Performance for Standard and Robust Linear Models

| Model | AIC | AIC Weight | BIC | RMSE | Sigma | *R*² | Adjusted *R*² |
| --- | --- | --- | --- | --- | --- | --- | --- |
| Robust Linear Model | 9165.3 (0.236) | 9166.2 (0.236) | 9318.4 (0.236) | 3.247 | 3.272 | — | — |
| Standard Linear Model | 9162.9 (0.764) | 9163.9 (0.764) | 9316.1 (0.764) | 3.245 | 3.27 | 0.151 | 0.138 |

*Note.* An em dash (—) indicates a value is not applicable for that model. *AIC* = Akaike Information Criterion; *AICc* = Corrected Akaike Information Criterion; *BIC* = Bayesian Information Criterion. The value in parentheses (*w*) represents the Akaike weight, indicating the relative likelihood of the model. RMSE = Root Mean Square Error; Sigma = Residual Standard Error; *R*² = R-squared.
